# Assessing intervention effects on healthcare-related financial protection in low- and middle-income countries: a rapid evidence review

**DOI:** 10.1093/heapol/czag065

**Published:** 2026-05-12

**Authors:** Krista Kruja, Nouria Brikci, Maria Paola Bertone, Sophie Witter

**Affiliations:** Evidence Link, Toronto, Ontario, Canada; NBDev Consulting, London, United Kingdom; Department of Global Health and Development, London School of Hygiene and Tropical Medicine, 15-17 Tavistock Pl, London WC1H 9SH, United Kingdom; Institute for Global Health and Development, Queen Margaret University, Queen Margaret University Drive, Edinburgh EH21 6UU, United Kingdom; ReBUILD for Resilience Consortium, C/O Liverpool School of Tropical Medicine Pembroke Place, Liverpool L3 5QA, United Kingdom; Institute for Global Health and Development, Queen Margaret University, Queen Margaret University Drive, Edinburgh EH21 6UU, United Kingdom; ReBUILD for Resilience Consortium, C/O Liverpool School of Tropical Medicine Pembroke Place, Liverpool L3 5QA, United Kingdom

**Keywords:** health financing, health systems, financial protection

## Abstract

Financial protection is a key goal for universal health coverage, but globally, this indicator has been lagging, especially for low-income households. Understanding how to improve it is urgent. This rapid evidence review examined the effectiveness of interventions, which affected financial protection in health across low- and middle-income countries (LMICs). A systematic search of published literature identified empirical studies reporting on outcomes such as out-of-pocket expenditure (OOPE), catastrophic spending, impoverishment, and care foregone for financial reasons, with data extracted and categorized using structured tools. A narrative synthesis was used to interpret the evidence. The review included 214 studies published between 1999 and 2024, covering interventions in 39 countries. About one-quarter of studies assessed equity, often revealing mixed or regressive effects. Financial protection is affected through multiple channels, but evaluations still largely focus on health-sector-specific interventions, failing to examine potentially impactful wider policies. Insurance schemes were the most studied intervention type, with mixed results. Demand-side financing, such as vouchers or cash transfers, and user fee reforms often struggled to benefit the poorest due to structural barriers and indirect costs. Provider payment reforms were more likely than other categories to show adverse effects, including unintended supplier behaviour changes that drove up OOPE and costs to the system. Less frequently evaluated interventions, such as social protection and behaviour change programmes, showed some positive effects. Whilst no single intervention consistently ensured financial protection, the evidence points to the value of interventions that act across multiple sectors and health areas. Designing reforms with an explicit objective of improving financial protection, with local context, system capacity, and equity in mind and adjusting based on continuous monitoring, can support achievement of improved and equitable financial protection.

Key messages

- No single intervention consistently ensures financial protection, which is a multi-sectoral outcome. Health-sector-specific interventions, such as insurance or fee removals, often fail because they do not address the broader structural barriers that keep households vulnerable. Governments must adopt multi-pronged, context-specific strategies that address health system factors, broader social determinants, and social protection.
- Financial protection is affected through multiple channels, but evaluations still largely focus on ‘classic’ health financing interventions such as insurance, neglecting to study potentially impactful wider policies.
- Policymakers should make financial protection an explicit objective and ensure it is monitored in a disaggregated way.

## Introduction

With development funding under pressure and domestic health budgets tightening, countries face growing difficulty in sustaining progress on universal health coverage. Financial protection in health, defined as ‘the absence of financial barriers to accessing needed care and lack of financial hardship due to out-of-pocket health spending’ (World Health Organisation and International Bank for Reconstruction and Development 2021), is essential to achieving global health goals. It is also one of two key indicators at the heart of Sustainable Development Goal (SDG) 3.8 [SDG indicator 3.8.2 is measured as the proportion of the population with OOP health spending exceeding 10% and 25% of total household expenditure or income, and the total population with impoverishing health spending includes people impoverished (pushed below the poverty line) and further impoverished (already below the poverty line but pushed even further below) due to OOP health spending.]. However, despite global commitments, close to one in four people globally will continue to face health-related financial hardship in 2030, the endpoint of the SDGs. Clear guidelines and policy recommendations are needed to ensure governments are able to prioritize financial protection in their reform agenda. However, which interventions work for financial protection is not clear from the current literature.

A scoping overview of studies published between 1990 and 2020 examined how financial protection has been conceptualized and assessed in the context of universal health coverage (Bhatia et al. 2022). Only half of the included reviews defined financial protection. It was commonly measured using out-of-pocket, catastrophic, and impoverishing health expenditures, and the main interventions studied for achieving financial protection were pooling arrangements, expansion of insurance coverage, and financial incentives. Evidence gaps pertained to the effectiveness, cost-effectiveness, and equity implications of efforts aimed at increasing financial protection.

Previously published reviews have examined the effects of specific interventions on financial protection in low- and middle-income countries (LMICs), such as community-based health insurance (CBHI) or micro-health insurance (Habib et al. 2016, Eze et al. 2023), publicly organized and financed health insurance (Erlangga et al. 2019), reduced user fees and charges (Qin et al. 2019), or hospital payment reforms (Ghazaryan et al. 2021). However, none reviewed impacts on financial protection from the whole range of possible interventions.

This paper presents findings from a rapid evidence review to address the urgent need for effective strategies to improve financial protection from health spending in LMICs. It focuses on the question: which policy choices or interventions in the health sector and beyond are most (or least) effective in improving financial protection in healthcare, drawing from published literature?

## Materials and methods

This study employed a rapid evidence review methodology. The Cochrane Collaboration defines a rapid review as ‘a form of knowledge synthesis that accelerates the process of conducting a traditional systematic review through streamlining or omitting specific methods to produce evidence for stakeholders in a resource-efficient manner’ (Garritty et al. 2021).

Searches were conducted between May and December 2024 in PubMed (including PMC, Medline, NCBI Bookshelf), Cochrane Library, and Web of Science, with no restrictions. We did not search trial registries or include unpublished studies.

The review aimed to identify published, empirical evidence about the effectiveness of policies and interventions in improving healthcare-related financial protection in LMICs. The search terms and inclusion criteria were designed to capture any study reporting on financial protection outcomes. Studies were then categorized by intervention type. This strategy was adopted to minimize selection bias by avoiding the *a priori* restriction of intervention types to those already known or assumed to affect financial protection.

The study selection process involved screening titles and abstracts of deduplicated records using Rayyan software, followed by full-text review of potentially eligible studies by two reviewers using Covidence software. Approximately 5% of records were randomly selected to be screened by two researchers to ensure consistency. Disagreements were resolved through discussion and consensus.

Empirical, primary research studies reporting results from LMICs were included. We excluded reviews and studies that did not assess any outcomes related to financial protection. Studies were only included if they were written in English, French, or Spanish.

**Figure 1:**
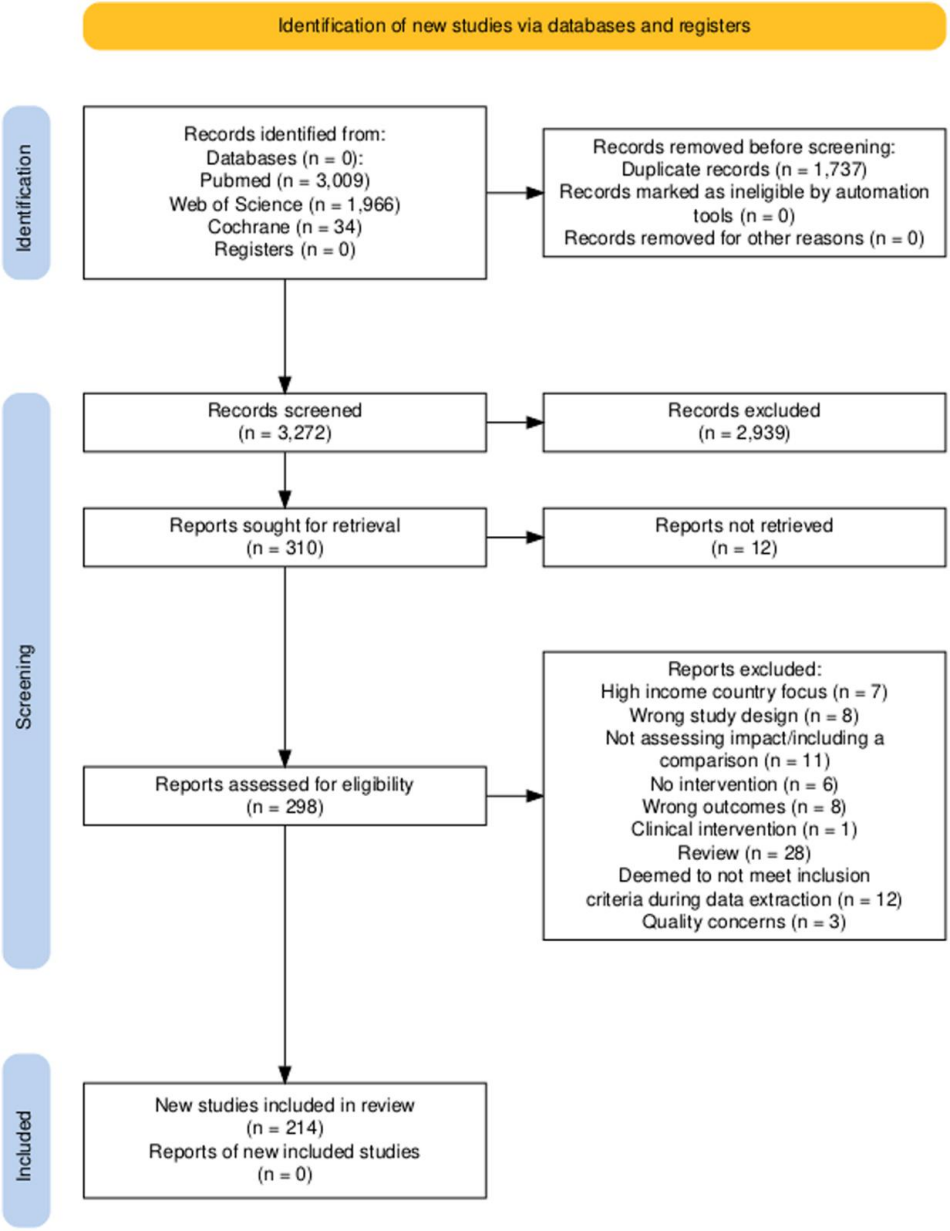
PRISMA diagram.

Search terms, inclusion and exclusion criteria are presented in Table 1. The Preferred Reporting Items for Systematic reviews and Meta-Analyses (PRISMA) diagram showing the study selection process is included as Fig. 1.

**Table 1:** Search terms and inclusion criteria.

Component	Area of interest	Search terms	Include studies which:	Exclude studies which:
**Population**	LMICs	n/a	Provide evidence from an LMIC or a group of LMICs	Provide evidence from high-income countries only
**Intervention**	Interventions that may improve healthcare-related financial protection	n/a	Focus on an intervention that may affect financial protection–related indicators	Do not assess any outcomes related to financial protection
**Comparison**	Any comparison, including no intervention/baseline	‘reform’ OR ‘policy’ OR ‘programme*’ OR ‘intervention’ OR ‘impact’ OR ‘evaluation’ OR ‘trial’ OR ‘pilot’ OR ‘trial’ OR ‘Review’ OR ‘effect*’ OR ‘Efficacy’	Empirically assess the effects of an intervention using observational or experimental methods	Do not include an intervention
**Outcome**	Outcomes relating to financial protection	‘Financial protection’ OR ‘Financial risk protection’ OR ‘Impoverish*’ OR ‘Out of pocket’ OR ‘out-of-pocket’ OR ‘Catastrophic health expenditure’ OR ‘financial hardship’ OR ‘Foregone care’ OR ‘Coping strategies’ OR ‘User fees’ OR ‘Consumption’ OR ‘Savings’ AND ‘’healthcare’’ [and synonyms]	Examine the effects of an intervention on at least one indicator of financial protection	Do not examine intervention effects on financial protection

### Data extraction and analysis

Data from included studies were extracted using a structured data extraction sheet, capturing information on study details, country/region, study design, intervention characteristics, intervention approach (setting, scale, duration, funding), quality appraisal criteria, impact on primary outcomes (relating to financial protection), impact on secondary outcomes (equity, intervention costs, cost-effectiveness, adverse outcomes), and factors affecting design and implementation (political, economic, bureaucratic, other), where they were reported by the study. The extraction process was carried out in Covidence by multiple researchers, with a subset of studies quality-assessed by two researchers to ensure consistency.

An inductive approach was adopted to group studies for analysis based on the category of intervention. Intervention categories were defined based on how interventions were described and studied in the included literature. Where a study evaluated multiple distinct intervention types or components, each was treated as a separate analytical record. We classified interventions according to the most direct mechanism of action being evaluated by study authors and noted where a secondary intervention type was also relevant.

Reported outcomes were categorized as out-of-pocket expenditure (OOPE), catastrophic health expenditure (CHE), impoverishment, service use, equity, or other. These outcomes are defined and described in Table 2. Each reported outcome was individually classified as beneficial, harmful, or no change. If a change was not statistically significant, it was categorized as no change (except for equity outcomes). Equity outcomes were assessed on a case-by-case basis due to variability in reporting.

**Table 2:** Description of outcomes.

Outcome	Description
**OOPE**	OOPE in healthcare refers to the direct costs borne by patients or their families for medical services, treatments, and related expenses, typically excluding insurance reimbursements but potentially encompassing direct non-medical costs such as transportation and food. The types of costs included also varied, with some studies focusing solely on direct medical costs, while others included non-medical expenses (like transportation) or even indirect costs (such as lost wages). The treatment of insurance reimbursements differed as well, with some studies considering payments made at the point of service (co-payments, deductibles) while others looked at net costs after reimbursements. The level of analysis ranged from individual to household or per capita levels, with some studies narrowing their focus to specific conditions (e.g. hospitalization, cancer treatment) while others assessed all healthcare services. These methodological variations highlight the complexity in comparing OOPE outcomes across studies.
**CHE**	CHE occurs when healthcare expenses surpass a certain proportion of a household's financial capacity. The studies included in this review use various methods to measure CHE, differing in thresholds, definitions of capacity to pay, healthcare costs considered, and assessment metrics. Differences in thresholds: The most common threshold used is 40% of a household's capacity to pay, defined as total consumption expenditure minus food costs. This focuses on discretionary spending, where healthcare expenses exceeding this threshold are considered catastrophic. Alternatively, studies also used a 10% threshold of total household income, without adjusting for essential (food) expenditures, offering a broader perspective, or explored thresholds ranging from 20% to 60%, though typically as part of a sensitivity analysis. Differences in the denominator (definition): Studies used a range of denominators: they used total household consumption minus food expenses to define capacity to pay, relied on regional metrics like county-level rural per capita income adjusted for household size, or used annual disposable income excluding taxes. Differences in the numerator (OOPE): Generally, studies used OOPE as the numerator. As a result, methodological variations noted in the row above for OOPE add an additional layer of variation to how CHE is calculated. Differences in metrics used: Metrics include CHE incidence (percentage of affected households), prevalence (proportion within a population), and mean catastrophic payment gap (severity of financial burden). Studies also evaluated protection from CHE by examining changes in incidence after insurance reimbursements.
**Impoverishment**	Differences in how this is measured often stem from variations in defining the poverty line and the specific metrics used. Key measures include the incidence of poverty (whether a household's income per capita falls below the poverty line), extreme poverty (often based on World Bank thresholds), and the depth or severity of poverty (how far below the poverty line a household falls). Studies also estimated the vulnerability to poverty, or the probability that a household will become poor due to health expenses. Additionally, the same metric may be applied in different ways. For example, poverty depth can be assessed by measuring the income shortfall of poor households relative to the poverty line after spending on healthcare, or alternatively, by evaluating whether OOPE exceed a certain proportion of annual household income.
**Foregone care/service utili**z**ation**	Healthcare utilization was included as an outcome as several studies interpret foregone or reduced care as evidence of inadequate financial protection. The studies included in the systematic review use a variety of approaches to measure healthcare service utilization and foregone care, focusing on both the type of care accessed and the barriers that prevent individuals from receiving needed services. Metrics such as delayed care, self-treatment, unmet needs for treatment (e.g. foregone hospitalization when needed), and partial treatment (e.g. early hospital discharges due to cost concerns) are used to capture instances where patients forgo necessary care. Studies assessing service utilization examined inpatient care (e.g. hospital admissions and length of stay) and outpatient visits, using metrics such as the number of visits, admissions, and the use of specific services like preventive care, dental services, or rehabilitation. Utilization data are collected through various sources, including patient self-reports (via surveys), health service and admission records, and financial indicators such as total healthcare expenditure. Additionally, some studies explore shifts in the use of different types of care, such as traditional versus formal medical care, or primary versus tertiary care. However, when these shifts are reported without clarifying whether they had a beneficial impact on access or use of healthcare services, these changes are only included in the narrative results rather than in the tables of findings.
**Equity**	Some studies explicitly measure equity as an outcome, while others report disaggregated results across groups, allowing for inferences about changes in equity. Direct measures include the concentration index (CI), which assesses how healthcare utilization or financial burdens, like OOPE and CHE, are distributed among income groups. The CI helps identify whether interventions reduce disparities. Some studies use the Gini coefficient to evaluate income equity, particularly in rural areas. Disaggregated analyses compare outcomes across socioeconomic groups, such as income quartiles, urban versus rural populations, age groups, gender, and those living in poverty versus those not.
**Other outcomes**	Several studies assessed alternative indicators of financial protection, beyond standard measures. These included outcomes such as changes in household savings and the proportion of medical expenses financed through distress financing mechanisms, such as selling assets or borrowing, rather than relying on income, savings, or other stable sources. Other outcomes were used as proxies for the uptake of interventions aimed at improving financial protection. For example, enrolment in SHI schemes was used to assess the effect of reducing registration costs. Some studies also examined outcomes related to the cost and financial sustainability. A small number of studies also reported outcomes not directly related to answering our research question, and these were only extracted if they reflected adverse consequences of the intervention. Relevant examples included mortality, health outcomes, service quality (e.g. waiting times), and provider behaviour.

Studies were assessed for quality using the Mixed Methods Appraisal Tool (Hong et al. 2018). Three studies were excluded due to methodological weaknesses, including unclear causal links between interventions and outcomes or inappropriate use of data and methods. All studies that were not removed due to quality concerns were treated with equal weight in drawing conclusions. While the quality of individual studies varied, their collective inclusion was guided by the principle of triangulation, leveraging diverse methodologies to paint a more comprehensive picture of intervention effects.

A narrative synthesis approach was employed to interpret intervention effects by examining the breadth of evidence and key contextual insights from individual studies. This approach highlighted common patterns, inconsistencies, trade-offs, underlying mechanisms, and illustrative examples, accounting for nuances across populations, settings, and outcome measures.

## Results

### Overview of included studies

This review included 214 studies, representing 231 records, conducted between 1999 and 2024, covering interventions across 39 countries. Included studies are summarized in Supplementary Annex S1. Only one study focused on multiple countries, while all others examined interventions within a single nation. China emerged as the most frequently studied country, with 101 records (96 studies). The majority of records (*n* = 163) analysed interventions implemented at the national level, while the remainder focused on pilot programmes or interventions at subnational levels.

Most studies (∼70%) were observational, ∼25% were quasi-experimental and only 11 studies (∼5%) used experimental methodologies, such as cluster-randomized controlled trials (Thornton et al. 2010, Fink et al. 2013, Grogger et al. 2015, Dror et al. 2016, Raza et al. 2016, Sunny et al. 2021, Choudhary et al. 2022, Koch et al. 2022, Rabbani et al. 2022, Mori et al. 2024, Tsuei and Yip 2024). The included studies used a variety of research methodologies to assess the impact of programmes and policies.

All studies evaluated financial protection using one or more of four primary outcome domains: OOPE, CHE, impoverishment, and service use or foregone care. Table 2 provides further details of what each outcome category captures and the methodological variation in how they are measured and analysed.

Seventy-three per cent of records assessed OOPE. This was followed by changes to service use (51%) and CHE (40%). Impoverishment was the least frequently assessed outcome in only 17% of records. Equity was assessed in 28% of studies. Several studies also examined other impacts of financial protection interventions, such as changes in unmet needs for medicines, service substitution effects (e.g. from private to public care facilities), and the impact on income losses due to illness or illness-related caring duties. A small proportion of studies (∼5%) also reported adverse consequences outside of the primary outcome categories examined, such as worsened quality or supplier-induced demand. These are reported in the intervention-specific analyses below.

Evidence on cost of care and cost-effectiveness is limited. Only 30 studies reported such data, with variable quality and few formal analyses.

### Intervention effects on health-related financial protection

The groupings used to analyse intervention effects are presented in Table 3. Studied interventions were categorized as follows: ‘demand-side financing’ [9 records (Shi et al. 2010, Gopalan and Durairaj 2012, Nguyen et al. 2012a, Obare et al. 2015, Liu et al. 2017, Mukherjee and Singh 2018, Nannini et al. 2021, Pan et al. 2022, Chen et al. 2023a)], ‘user fee reforms’ [15 records (Lambert-Evans et al. 2009, Caballes et al. 2012, Tripathi et al. 2014, Ridde et al. 2015, Edoka et al. 2016, Masiye et al. 2016, Witter et al. 2016, Neelsen and O’Donnell 2017, Lépine et al. 2018, Korachais et al. 2019, Dickerson et al. 2020, Bousmah et al. 2021, Sunny et al. 2021, van Duinen et al. 2021, Aye et al. 2023)], ‘behaviour change’ [1 record (Choudhary et al. 2022)], ‘health facility upgrades’ [1 record (Bonfrer et al. 2018)], ‘shift in service delivery organization and funding’ [7 records (Homaie Rad et al. 2017, Zhang et al. 2017, Tirgil et al. 2019, Erus 2020, Fontanet et al. 2021, Peresu et al. 2024, Zhu et al. 2024)], ‘provider payment reforms’ [21 records (Erus and Aktakke 2012, Gao et al. 2014, Binyaruka et al. 2015, Anselmi et al. 2017, He et al. 2017, Li et al. 2018, Jiang et al. 2019b, Li et al. 2019b, Meng et al. 2019a, 2022, Xin et al. 2019, Damrongplasit and Atalay 2021, Thein et al. 2021, Wu et al. 2022, Zhang et al. 2022, Aye et al. 2023, Chen et al. 2023b, Zhang et al. 2023b, Tsuei and Yip 2024, Xiang et al. 2024)], ‘reducing cost and increasing access to essential medicine’ [15 records (Honda and Hanson 2013, Yu et al. 2013, Qiu et al. 2014, Sun et al. 2015, Chen et al. 2017, Ding and Wu 2017, Bose and Dutta 2018, Wang et al. 2019, Chen et al. 2020, Cai et al. 2022, Diao et al. 2022, Zhu et al. 2022, Chu et al. 2023, Liu et al. 2023b, Yang et al. 2023)], ‘social protection’ [4 records (Riumallo-Herl and Aguila 2019, Li et al. 2023b, Wang et al. 2023, Mori et al. 2024)], and ‘insurance and related interventions’.

**Table 3:** Studies by intervention category.

Intervention category	Description	Included records	Citations
Demand-side financing	Interventions targeted demand-side elements through various means, including vouchers, conditional cash transfers, medical reimbursements, insurance subsidies, and zero-interest loans. Demand-side financing reforms included voucher programmes in Bangladesh and Kenya, both targeting pregnant women and delivery, and for maternity care in India; direct reimbursement of medical expenditures in China; subsidization of insurance premia for low-income households in China and the Philippines; and a zero-interest health loan to support medical expenditures in Uganda (see Table 3). Most of the studied interventions in this category targeted vulnerable groups such as the poor, elderly, disabled, and/or mothers/pregnant people. The exceptions were the Medical fee direct reimbursement in China (Pan et al. 2022) and zero-interest health loans in Uganda (Nannini et al. 2021).	9	Shi et al. 2010, Gopalan and Durairaj 2012, Nguyen et al. 2012a, Obare et al. 2015, Liu et al. 2017, Mukherjee and Singh 2018, Nannini et al. 2021, Pan et al. 2022, Chen et al. 2023a
User fee reforms	User fee reforms included abolition, reduction, as well as exemptions. Seven countries removed user fees for some or all parts of care for pregnant women and their newborn, at times children up to 5 (Sierra Leone, India, Benin, Burkina Faso, Mali, Morocco, and Nepal). Burundi, Cambodia, and Peru removed user fees for poor sections of the population. Cameroon and Malawi removed user fees for people living with HIV (PLHIV), in particular for the antiretroviral therapy (ART), and Zambia removed user fees for all PHC level services.	15	Lambert-Evans et al. 2009, Caballes et al. 2012, Tripathi et al. 2014, Ridde et al. 2015, Edoka et al. 2016, Masiye et al. 2016, Witter et al. 2016, Neelsen and O’Donnell 2017, Lépine et al. 2018, Korachais et al. 2019, Dickerson et al. 2020, Bousmah et al. 2021, Sunny et al. 2021, van Duinen et al. 2021, Aye et al. 2023
Behaviour change	Only one study specifically assessed the effects of a behaviour change intervention piloted to address maternal health in India.	1	Choudhary et al. 2022
Upgraded health facilities	Only one study assessed the specific effects of upgrading health facilities (Bonfrer et al. 2018). The Kwara state of Nigeria upgraded health infrastructure alongside the establishment of a VHI mechanism.	1	Bonfrer et al. 2018
Shift in service delivery organization and funding	Five countries refocused their service delivery on primary healthcare: Iran empowered GPs to lead PHC teams whilst integrating a strict referral and gatekeeping system; Türkiye took a similar approach but focused on setting up Family Health Centres to provide preventive and primary care; Zambia established maternity waiting homes for new mothers; and finally, China piloted a hospital reform programme aimed at improving the cost-effectiveness of its spending. Lastly, Medecins Sans Frontieres implemented a MDR-TB patient-centred home-based treatment in Eswatini.	7	Homaie Rad et al. 2017, Zhang et al. 2017, Tirgil et al. 2019, Erus 2020, Fontanet et al. 2021, Peresu et al. 2024, Zhu et al. 2024
Provider payment reforms	Provider reforms were numerous in China. Different areas of the country experimented with case-based payments, capitation, diagnostic related groups (DRG), diagnosis intervention packet (DIP), episode bundled payments (EBPs), quota payments within global budgets, and global budgets. Documented reforms in other countries are less complex, including the introduction of capitation in Myanmar, pay for performance in Tanzania, DRGs in Thailand, and pay for performance and contracting in Türkiye.	21	Erus and Aktakke 2012, Gao et al. 2014, Binyaruka et al. 2015, Anselmi et al. 2017, He et al. 2017, Li et al. 2018, Jiang et al. 2019b, Li et al. 2019b, Meng et al. 2019a 2022, Xin et al. 2019, Damrongplasit and Atalay 2021, Thein et al. 2021, Wu et al. 2022, Zhang et al. 2022, Aye et al. 2023, Chen et al. 2023b, Zhang et al. 2023b, Tsuei and Yip 2024, Xiang et al. 2024
Reducing cost and increasing access to essential medicine	Reforms related to reducing cost and increasing access to essential medicine have been documented in three countries: China, where the government established an essential drugs list on which a zero mark-up policy was imposed to providers; India, where a list of medicines was either fully subsidized or partially subsidized in several states; and Madagascar, where the resources collected through an equity fund were used to subsidize medicines. Studies on drug subsidy interventions evaluated the effects of covering specific drugs or categories through reimbursement lists in China (Qiu et al. 2014, Cai et al. 2022, Diao et al. 2022, Liu et al. 2023b, Yang et al. 2023), broad-scope medicine coverage policies in China (Yu et al. 2013, Sun et al. 2015, Chen et al. 2017, 2020, Wang et al. 2019, Zhu et al. 2022, Chu et al. 2023), and medicine subsidies targeting the poor in India (Bose and Dutta 2018) and Madagascar (Honda and Hanson 2013).	15	Honda and Hanson 2013, Yu et al. 2013, Qiu et al. 2014, Sun et al. 2015, Chen et al. 2017, Ding and Wu 2017, Bose and Dutta 2018, Wang et al. 2019, Chen et al. 2020, Cai et al. 2022, Diao et al. 2022, Zhu et al. 2022, Chu et al. 2023, Liu et al. 2023b, Yang et al. 2023
Social protection	Three social protection reform, all utilizing cash transfers, were identified: Reconocer pension programme in Mexico, using a cash transfer mechanism to augment recipient's food baskets and income, a cash transfer mechanism in Zambia, aimed at promoting a wide range of social benefits, and a cash transfer in China aimed at supporting disabled adults and their carers.	4	Riumallo-Herl and Aguila 2019, Li et al. 2023b, Wang et al. 2023, Mori et al. 2024
Insurance and related interventions	Insurance reforms were by far the most widely studied (in 22 countries, across 139 records). Efforts to increase population coverage took time, as is apparent by the evolution of reforms we see for example in China or Vietnam, which have both started with fragmented schemes for the formal sector and slowly moved to include poor segments of the population, the informal sector, or otherwise vulnerable population groups such as children or students. Other countries have also worked at increasing population coverage through including the informal sector (e.g. women in the ready-made garment industry in Bangladesh) and poor segments of the population (e.g. people living below the poverty line in India and Bangladesh). The modalities for the insurance schemes varied in each country and evolved over time: some chose to rely on some form of VHI schemes as the key building block for their coverage extension (e.g., Burkina Faso or Ethiopia), whilst others chose to rely on PFHI schemes, which are targeted towards poor, marginalized, or vulnerable populations (e.g., Georgia or Mexico), or SHI, which targets the broader population (e.g., Ghana or Vietnam), although nearly all countries identified used a combination of mechanisms to progress coverage. The expansion of the benefit package was used as a reform to improve financial protection in India (inclusion of MDR-TB), Kenya (inclusion of NCD services), and China (various reforms to increase inpatient and outpatient services included in the various insurance schemes' benefit packages). China also relied on improving the level of compensation of the insured population through each of its health insurance schemes.	139	See breakdown below
PFHI		*30*	Bauhoff et al. 2011, Sosa-Rubí et al. 2011, Caballes et al. 2012, Fan et al. 2012, Nguyen et al. 2012b, Aji et al. 2013, Sood et al. 2014, Yardim et al. 2014, Gotsadze et al. 2015a, 2015b, Grogger et al. 2015, Nguyen 2016, Knaul et al. 2018, Martínez-García et al. 2018, Nikoloski and Mossialos 2018, Ranjan et al. 2018, Celhay et al. 2019, Garg et al. 2019, Tirgil et al. 2019, Garg et al. 2020, Sriram and Khan 2020, Maroof and Sangmi 2021, Garg et al. 2022a, 2022b, Ly et al. 2022, Garcia-Diaz et al. 2023, Parmar et al. 2023, Serván-Mori et al. 2023, Hasan et al. 2024
SHI		*55*	Hu et al. 1999, Somkotra and Lagrada 2008, Axelson et al. 2009, Lei and Lin 2009, Sun et al. 2009, Wagstaff et al. 2009, Babiarz et al. 2010, Shi et al. 2010, Nguyen et al. 2011, Caballes et al. 2012, Fang et al. 2012, Lu et al. 2012, Nguyen et al. 2012b, 2023, Aji et al. 2013, Nguyen and Wang 2013, Sparrow et al. 2013, Abrokwah et al. 2014, Wang et al. 2014, 2018, Atella et al. 2015, Aryeetey et al. 2016, Brugiavini and Pace 2016, Guo et al. 2016, Ma et al. 2016, 2021, Sun et al. 2016, 2021, Zhang et al. 2016, Gu et al. 2017, Liu et al. 2017, 2023a, Zhou et al. 2017, Xiong et al. 2018, Fiestas Navarrete et al. 2019, He and Nolen 2019, Kanmiki et al. 2019, Karunaratna et al. 2019, Nguyen and Lo Sasso 2019, Tan et al. 2019, Nguyen 2020, Okoroh et al. 2020, Thuong et al. 2020, Frimpong et al. 2021, Jiang et al. 2021, Peng and Zhu 2021, Sarkodie 2021, Taverne et al. 2021, Wu and Ercia 2021, Zhai et al. 2021, Koch et al. 2022, Rabbani et al. 2022, Yang et al. 2022, Xue and Witvorapong 2023, Aashima and Sharma 2024
VHI		*25*	Jowett et al. 2003, Aggarwal 2010, Thornton et al. 2010, Fang et al. 2012, Nguyen et al. 2012b, Fink et al. 2013, Alkenbrack and Lindelow 2015, Savitha and Kiran 2015a, 2015b, Dror et al. 2016, Nguyen 2016, Raza et al. 2016, Bonfrer et al. 2018, Mebratie et al. 2019, Ahmed et al. 2020, Thuong et al. 2020, Peng and Zhu 2021, Wu and Ercia 2021, Bousmah et al. 2022, Chen and Ning 2022, Ly et al. 2022, Okunogbe et al. 2022, Ma and Xu 2023, Aashima and Sharma 2024, Ng et al. 2024
Insurance integration/overarching assessment of multiple schemes		*8*	Ruiz et al. 2007, Bodhisane and Pongpanich 2019, Wang et al. 2020, Bodhisane and Pongpanich 2022, Zhou et al. 2022, Ahmed and Mahapatro 2023, Huo et al. 2023, Aashima and Sharma 2024
Expanded benefit package or enhanced compensation		*21*	Jing et al. 2013, Yuan et al. 2014, Li et al. 2015, Yang and Wu 2015, Dai et al. 2016, Xiang et al. 2016, You et al. 2016, Kundu et al. 2018, Miao et al. 2018, Jiang et al. 2019a, Li et al. 2019a, 2019c, 2019d, 2020, Zhao et al. 2020, Yu et al. 2021, Zhang et al. 2021, 2023a, Zhong et al. 2021, Cao et al. 2022, Oyando et al. 2023
Complex	Three countries stand out for having introduced reforms on both supply and demand side to improve financial protection: Cambodia, with a focus on a health equity fund, the introduction of user fees and voucher systems for women and children, CBHI and contracting reforms; Iran, with a refocus on PHC and family medicine, a reduction of OOPE, and a programme of physician retention; and China, through a wide ranging healthcare reform, supported by general and targeted poverty alleviation programmes focused on a combination of infrastructure investment, health insurance, and assistance for the most vulnerable.	19	Ensor et al. 2017, Assari Arani et al. 2018, Huang et al. 2018, Xu et al. 2018, Ahmadnezhad et al. 2019, Chen and Pan, 2019, Joshani Kheibari et al. 2019, Xu et al. 2019, Nemati et al. 2020, Darvishi et al. 2021, Esmaeili et al. 2021, Liu et al. 2021, Ahmadnezhad et al. 2023, Huang et al. 2023, Li et al. 2023a, Malekroudi et al. 2023, Mohammadzadeh et al. 2023, Tang et al. 2023, Cui et al. 2024)

Insurance-related interventions were analysed by sub-categories depending on whether they examined ‘Social Health Insurance (SHI)’ [55 records (Hu et al. 1999, Somkotra and Lagrada 2008, Axelson et al. 2009, Lei and Lin 2009, Sun et al. 2009, Wagstaff et al. 2009, Babiarz et al. 2010, Shi et al. 2010, Nguyen et al. 2011, Caballes et al. 2012, Fang et al. 2012, Lu et al. 2012, Nguyen et al. 2012b, 2023, Aji et al. 2013, Nguyen and Wang 2013, Sparrow et al. 2013, Abrokwah et al. 2014, Wang et al. 2014, 2018, Atella et al. 2015, Aryeetey et al. 2016, Brugiavini and Pace 2016, Guo et al. 2016, Ma et al. 2016, 2021, Sun et al. 2016, 2021, Zhang et al. 2016, Gu et al. 2017, Liu et al. 2017, 2023a, Zhou et al. 2017, Xiong et al. 2018, Fiestas Navarrete et al. 2019, He and Nolen 2019, Kanmiki et al. 2019, Karunaratna et al. 2019, Nguyen and Lo Sasso 2019, Tan et al. 2019, Nguyen 2020, Okoroh et al. 2020, Thuong et al. 2020, Frimpong et al. 2021, Jiang et al. 2021, Peng and Zhu 2021, Sarkodie 2021, Taverne et al. 2021, Wu and Ercia 2021, Zhai et al. 2021, Koch et al. 2022, Rabbani et al. 2022, Yang et al. 2022, Xue and Witvorapong 2023, Aashima and Sharma 2024)], ‘Publicly Funded Health Insurance (PFHI)’ [30 records (Bauhoff et al. 2011, Sosa-Rubí et al. 2011, Caballes et al. 2012, Fan et al. 2012, Nguyen et al. 2012b, Aji et al. 2013, Sood et al. 2014, Yardim et al. 2014, Gotsadze et al. 2015a, 2015b, Grogger et al. 2015, Nguyen 2016, Knaul et al. 2018, Martínez-García et al. 2018, Nikoloski and Mossialos 2018, Ranjan et al. 2018, Celhay et al. 2019, Garg et al. 2019, 2020, Tirgil et al. 2019, Sriram and Khan 2020, Maroof and Sangmi 2021, Garg et al. 2022a, 2022b, Ly et al. 2022, Garcia-Diaz et al. 2023, Parmar et al. 2023, Serván-Mori et al. 2023, Hasan et al. 2024)], or ‘Voluntary Health Insurance (VHI)’ [25 records (Jowett et al. 2003, Aggarwal 2010, Thornton et al. 2010, Fang et al. 2012, Nguyen et al. 2012b, Fink et al. 2013, Alkenbrack and Lindelow 2015, Savitha and Kiran 2015a, 2015b, Dror et al. 2016, Nguyen 2016, Raza et al. 2016, Bonfrer et al. 2018, Mebratie et al. 2019, Ahmed et al. 2020, Thuong et al. 2020, Peng and Zhu 2021, Wu and Ercia 2021, Bousmah et al. 2022, Chen and Ning 2022, Ly et al. 2022, Okunogbe et al. 2022, Ma and Xu 2023, Aashima and Sharma 2024, Ng et al. 2024)].

SHI, also referred to as national health insurance, includes schemes targeting the whole population that are government-led and subsidized, but which include payments from individual or employers’ premia and are (in theory) compulsory. PFHI share some features of SHI, but are mostly voluntary and non-contributory or heavily subsidized, and usually aim to cover only a segment of the population, such as the poor (or informal sector). VHI included all voluntary schemes, such as CBHI, which may be owned by communities or other non-state actors, as well as private health insurance schemes. Additionally, 8 records assessed ‘multiple insurance schemes or integration of insurance schemes’ (Ruiz et al. 2007, Bodhisane and Pongpanich 2019, Wang et al. 2020, Bodhisane and Pongpanich 2022, Zhou et al. 2022, Ahmed and Mahapatro 2023, Huo et al. 2023, Aashima and Sharma 2024), and 21 records assessed ‘benefit package expansion or enhancement’ (Jing et al. 2013, Yuan et al. 2014, Li et al. 2015, Yang and Wu 2015, Dai et al. 2016, Xiang et al. 2016, You et al. 2016, Kundu et al. 2018, Miao et al. 2018, Jiang et al. 2019a, Li et al. 2019a, 2019c, 2019d, 2020, Zhao et al. 2020, Yu et al. 2021, Zhang et al. 2021, Zhong et al. 2021, Cao et al. 2022, Oyando et al. 2023, Zhang et al. 2023a). An additional category, ‘complex reforms’, was created for studies evaluating either multi-pronged or multiple interventions that could not be assigned to a single category.

These categories provide a useful organizing framework, but the interventions they each capture are heterogenous, driven by variation in intervention design, target populations, and implementation contexts. Findings for each category are presented below.

#### Demand-side financing and user fee reforms

Six of the nine demand-side financing interventions studied, such as vouchers or cash transfer, showed at least some beneficial effects on financial protection outcomes, including reductions in OOPE and CHE, improved utilization, and decreased reliance on harmful coping strategies. For instance, a voucher programme in Bangladesh reduced maternal OOPE by 640 Taka (US$9.43), equivalent to 64% of average monthly per capita household expenditure (Nguyen et al. 2012a). CHE incidence fell under China's intra-provincial medical reimbursement policy (Pan et al. 2022), and two demand-side financing schemes were associated with improved maternal care access in India (Gopalan and Durairaj 2012). A community-based pilot of zero-interest healthcare loans in Uganda reduced reliance on costly coping strategies (Nannini et al. 2021). Only one study of a demand-side financing intervention, a conditional cash transfer programme for deliveries in India, reported a harmful change; OOPE for institutional deliveries supported by the programme was around US$80, higher than for home deliveries (US$29–32) or even institutional births that were not financially supported by the programme (US$35) (Gopalan and Durairaj 2012). Patients incurred significant OOPE to travel to distant facilities eligible for the cash transfer, despite closer non-eligible options. This undermined the scheme's protective effect (Gopalan and Durairaj 2012).

Another study showed that poorer beneficiaries of the same cash transfer programme experienced slightly larger proportional reductions in OOPE than wealthier groups; they continued to spend more in absolute terms, indicating that pre-existing inequities in financial burden persisted (Mukherjee and Singh 2018). Structural and systemic barriers also limited scheme benefit in China, where the Medical Financial Assistance programme, aimed at low-income households, had limited success in reducing financial hardship (Shi et al. 2010, Liu et al. 2017).

User fee reforms similarly reduced OOPE, CHE, and inequities, but their impact was undermined by systemic issues that led to financial burdens in other areas. In several cases, informal payments, medicine costs, and other indirect expenses persisted or even increased. For example, in Cambodia, health expenditures decreased by 16.3% in intervention areas, while transport costs rose by 7% (Korachais et al. 2019). In Nepal, removing newborn care fees reduced costs for hospital beds but increased spending on medicines (Sunny et al. 2021). In India, despite fee exemption policies like Janani Shishu Suraksha Karyakram, OOPE remained high due to persistent shortages of drugs, diagnostics, and essential supplies in public facilities (Tripathi et al. 2014). These findings suggest that while demand-side financing and user fee reforms can improve financial protection, their effectiveness depends heavily on supply-side readiness, availability of services and commodities, and mechanisms to address indirect costs and informal charges.

#### Shift in service delivery organization and funding

Reorganizing service delivery [such as strengthening primary healthcare (PHC), hospital reforms, and switching to alternative care settings] showed mixed results for OOPE and service use outcomes, with minimal study of their effects on impoverishment and CHE. Four out of seven records in this category examined interventions targeting family medicine and PHC, which were studied in Iran (Homaie Rad et al. 2017), Türkiye (Erus 2020, Tirgil et al. 2023), and China (Zhu et al. 2024). In Iran, the family physician plan introduced an enhanced role for general practitioners in leading PHC teams and serving as gatekeepers for referrals. Similarly, in China, a general practitioner-led PHC workforce was established, alongside efforts to attract and retain qualified physicians through better remuneration, working conditions, and professional development. In Türkiye, the Family Medicine Programme engaged general practitioners as independent family doctors to expand PHC access. However, results across these interventions were mixed. In Türkiye, the programme increased the likelihood of incurring OOPE (Erus 2020), reduced monthly OOPE for doctor visits by 42% on average and by up to 122% in some regions (Tirgil et al. 2023). It also improved access, measured by increased per capita healthcare visits, and reduced inability to visit a physician (Erus 2020). In contrast, Iran's family physician plan had no significant impact on overall service use or OOPE. In both Iran and Türkiye, equity outcomes worsened: in Iran, the urban programme increased inequality, with the concentration index for drug services rising from 0.0072 to 0.707, indicating disproportionate benefits for wealthier individuals (Homaie Rad et al. 2017). In Türkiye, the financial burden grew more for poorer households, with a greater rise in healthcare spending among the lowest spenders (Erus 2020). In China, PHC strengthening significantly reduced household CHE and slightly lowered health expenses as a share of total consumption for both urban and rural residents (Zhu et al. 2024). No harmful results were reported, but equity outcomes were not assessed.

#### Provider payment reforms

Provider payment reforms included performance-based financing, capitation, and diagnostic-related groups. Similar to service delivery interventions, these reforms led to improved service use. However, only five studies in this category examined equity implications of the intervention, and none found beneficial equity effects; four of them reported harmful effects on equity (Jiang et al. 2019b, Xin et al. 2019, Chen et al. 2023b, Xiang et al. 2024) and one reported no change (Thein et al. 2021). For example, in Myanmar, a capitation-based health financing scheme was delivered through private general practice (GP) clinics, which were contracted to provide PHC services to poor households in their area (Thein et al. 2021). The scheme led to overall reductions in OOPE as a proportion of capacity to pay, but did not improve equity; poorer households continued to allocate a larger portion of their capacity to pay towards healthcare compared to wealthier households. Based on a very small sample size (6 people, or 0.7% of the study population), the scheme also significantly reduced healthcare-related impoverishment. In China, payment reforms led to worsened equity outcomes in several instances: case-based payment reforms increased the financial burden on lower-income patients with tuberculosis (Jiang et al. 2019b), transitioning from fee-for-service to diagnostic-related group payments in rural areas increased out-of-pocket costs and reduced service quality for rural residents, who already experienced inequities in healthcare and financial hardship (Xiang et al. 2024), and diagnosis intervention packages that did not adjust payments for age or gender (unlike standard diagnostic-related groups) led to rising out-of-pocket costs among the oldest-old hospitalized patients, increasing by 2.5% per month after reform (Chen et al. 2023b).

Effects on OOPE were mixed, with the same intervention types at times showing beneficial outcomes in some services and harmful outcomes in others. For example, in Tanzania, pay for performance schemes reduced OOPE for institutional deliveries, suggesting better enforcement of fee exemptions (Binyaruka et al. 2015, Anselmi et al. 2017). However, the same intervention showed weak evidence of increased OOPE for antenatal care and a higher likelihood of informal payments, though these findings were only marginally significant and varied depending on the analytical model used (Binyaruka et al. 2015). The effects also varied by specific interventions, context, and study design. For example, in China, capitation reduced inpatient OOPE (Gao et al. 2014), implementation of diagnostic-related groups decreased OOPE per admission and OOPE as share of inpatient expenditure (Meng et al. 2022), but a study that examined diagnostic-related groups combined with capitation found no impact on OOPE per inpatient visit (Zhang et al. 2023b).

Among provider payment reforms, there were more frequent reports of adverse consequences relating to reduced service quality or supplier-induced demand. In China, reforms that involved moving away from fee-for-service, including global budget reforms, quota payment systems, case-based payment reform for coronary heart disease, and introduction of an adapted diagnostic-related group mechanism, contributed to several adverse consequences, including increased hospital admissions and readmissions (He et al. 2017, Wu et al. 2022, Zhang et al. 2022) and increased length of hospital stay (Li et al. 2018). Studies also found evidence of cream-skimming behaviours by health providers, such as selecting younger patients and up-coding complications (Wu et al. 2022), and increased reimbursement under the NCMS following a switch to case-based payment for inpatient TB care, presumably because of compensatory behaviour by providers (Xin et al. 2019). The introduction of diagnostic-related groups for tertiary hospitals serving rural populations in China also found that the intervention increased OOPE as well as insurance fund expenditure, indicating an increased burden on both system and patients (Xiang et al. 2024).

#### Access to medicines

Interventions that aimed to address financial hardship related to medicine access were implemented primarily in China, as well as in India (Bose and Dutta 2018), and Madagascar (Honda and Hanson 2013) and operated through four key mechanisms: coverage of specific medicines, price negotiation, coverage or subsidies for specific populations, and a national policy, the National Essential Medicines System (NEMS) in China.

Coverage and price negotiations for cancer medicines and Hepatitis C therapies reduced OOPE (Cai et al. 2022, Diao et al. 2022, Liu et al. 2023b) and improved utilization for at least some target populations in all cases where it was examined (Qiu et al. 2014, Cai et al. 2022, Liu et al. 2023b, Yang et al. 2023). However, the effects of these interventions on CHE, impoverishment, and equity were not reported.

Broader reforms yielded mixed results. In Madagascar, equity funds had uneven impacts, with benefits for overall OOPE reduction observed in urban and suburban areas, but not in rural ones, raising concerns about equity (Honda and Hanson 2013). Additionally, the effect of the funds on medicine costs was limited, and significant reductions were observed only among suburban members.

The NEMS in China, examined in 11 studies, has 5 main objectives: establishing the National Essential Drugs List, sustaining quality standards, running the grassroots zero mark-up policy on essential medicines sales (only for Western Medicines), exempting herbal or Chinese-patented drugs, and improving rational drug use and public procurement. NEMS aimed to eliminate the profit link between physicians and the pharmaceutical industry and did not allow PHC providers to prescribe drugs outside of the essential medicines list. This programme was implemented by subnational authorities, and thus implementation varied by region. Whilst some studies indicate that the NEMS policy reduced OOPE (Yu et al. 2013, Wang et al. 2019, Chu et al. 2023), CHE (Sun et al. 2015), and impoverishment (Sun et al. 2015), others report increased OOPE. In Shanghai, NEMS raised OOPE and total spending for oesophageal cancer surgery inpatients (Chen et al. 2020), which researchers attributed to increases in prices of previously lower-cost medical processes and services to compensate for revenue lost due to drug mark-up withdrawal.

Similarly, a study in the Shandong region (Chen et al. 2017) distinguished between supply-side and demand-side elements of NEMS. The supply-side interventions required government-owned primary care providers to implement a zero mark-up policy for dispensing drugs, limiting them to those on the essential medicines list. This reduced drug prices but also constrained supply. The demand-side interventions focused on enhancing insurance coverage for medicines on the essential medicines list compared to those that were not, by adjusting social insurance benefits packages. Supply-side elements contributed to supplier-induced demand, leading to higher medicine costs and no significant reduction in overall healthcare expenditures. Costs were also higher at providers implementing both supply- and demand-side measures versus demand-side interventions alone. Drug expenditures surged by 88.6% during the supply-side ‘ramp-up’ period, ultimately increasing by 66.6% (*P* < 0.01) upon full implementation. One study also linked the NEMS policy to negative consequences for equity, showing that protection from impoverishment was more effective for richer households (third to fifth quintiles) than for poorer households (first and second quintiles) (Sun et al. 2015).

#### Insurance

Insurance schemes were the most frequently studied intervention category, examined in 138 studies, and showed substantial variation in design and outcomes. Insurance schemes differed in contribution models (e.g. who pays the premium, cost-sharing requirements), target populations, and benefit package design. In some countries, national schemes are implemented with significant local discretion. For example, China's Urban Employee Basic Medical Insurance (UEBMI), launched in 1998, provides mandatory coverage for urban employees, but local governments define benefit packages, set implementation timelines, and manage administration, resulting in wide regional variation (Liu et al. 2023a). While most schemes demonstrated some benefits, their effects were not uniformly positive across all populations or contexts.

Within insurance schemes, SHI, which targets the broader population, was the most studied category. The significant variation in reported results of these schemes were especially influenced by how heterogeneous SHI scheme designs were across and within countries. Whilst some aimed to cover the full national population, others were limited to specific groups. For example, SHI in Sri Lanka covered only civil servants (Karunaratna et al. 2019), and in Bangladesh covered those in formal employment (Rabbani et al. 2022). Many studies examined SHI in China, which was made up of multiple reforms that were implemented and iterated over time, each covering specific segments of the population: the UEBMI for formal sector urban employees, the NCMS for rural residents, and Urban Resident Basic Medical Insurance (URBMI), which aimed to cover residents not eligible for UEBMI. In 2016, the NCMS and URBMI were merged into a single scheme called the Urban and Rural Resident Basic Medical Insurance (URRBMI), with the aim of improving equity and administrative efficiency, but even URRBMI was not fully integrated nationally (Liu et al. 2023a). Subnational variation in terms of benefit design, reimbursement rates, and provider payment mechanisms across provinces and even municipalities also contributed to variation in outcomes (Ma et al. 2016, Zhang et al. 2016, Liu et al. 2023a, 2023b).

SHI schemes included a combination of supply and demand-side elements, and some evidence pointed to more positive outcomes when both were implemented together. For example, in Thailand, the SHI scheme was introduced alongside investments in public health facilities and quality assurance. An evaluation of the scheme reported positive effects on reducing impoverishment, the incidence of CHE, and inequities between poor and rich households (Somkotra and Lagrada 2008). In China, one study examined variation in outcomes across prefectures that adopted either demand-side reforms (focused on benefit expansion) or supply-side measures (focused on cost containment). It found that in areas without cost-containment policies, UEBMI significantly increased OOPE for individuals, including among enrolees, whereas in prefectures that implemented cost-containment reforms without expanding benefits, UEBMI reforms led to a significant increase in OOPE for uninsured individuals (Liu et al. 2023a).

Bureaucratic and administrative capacity also shaped how supply-side elements were implemented and, ultimately, the effectiveness of insurance schemes. In Ghana, delays in provider payments created tensions between resource-constrained providers and policies aiming to expand access for vulnerable groups (Fiestas Navarrete et al. 2019). Variation in management capacity and coordination mechanisms may help explain why similar interventions achieved different results across contexts. In China, limited managerial capacity was seen as a barrier to rural health insurance success (Li et al. 2015), while in Vietnam, the Free Care for Children Under Six scheme, which reduced OOPE and CHE and increased service use, required significant coordination across provincial health bureaus and government sectors, highlighting the role of strong administrative systems in effective implementation (Nguyen and Wang 2013).

Out of 14 studies of SHI where equity effects were reported, 8 studies reported at least some harmful effects (Wagstaff et al. 2009, Caballes et al. 2012, Lu et al. 2012, Guo et al. 2016, Gu et al. 2017, Wang et al. 2018, Ma et al. 2021, Yang et al. 2022). In these cases, SHI schemes improved healthcare use and financial protection more for better-off or urban groups, while poorer, rural, or informal sector populations at times received less protection or access, even when formally covered. For example, in China, wealthier households used more services and received greater reimbursement under UEBMI and NCMS, and inpatient care benefits were concentrated among richer groups (Wagstaff et al. 2009, Wang et al. 2018). In the Philippines, PhilHealth reimbursements were regressive: poorer patients received lower reimbursement despite higher need (Caballes et al. 2012). In Rwanda, households in the lowest expenditure quintile covered by Mutuelles had the highest probability of experiencing catastrophic health spending (Lu et al. 2012).

Among PFHI schemes, five out of seven records that reported on equity reported at least some harmful changes. These were in Mexico, for both the Seguro Popular programme and Instituto de Salud para el Bienestar, which replaced it (Knaul et al. 2018, Garcia-Diaz et al. 2023, Serván-Mori et al. 2023), Philippines (Caballes et al. 2012), and Senegal (Ly et al. 2022). However, Seguro Popular also showed beneficial equity outcomes, and the PFHI programme in Georgia led to reports of only beneficial or no change (Bauhoff et al. 2011).

Study authors highlighted implementation gaps, provider behaviour, or limited access to covered services as factors mediating the effects of various PFHI schemes. In Georgia, CHE incidence rose from 11.7% in 2007 to 24.8% in 2010 following the Medical Insurance for the Poor programme, which also shifted utilization towards costlier outpatient and private care (Zoidze et al. 2013). In India, the national PFHI programme Pradhan Mantri Jan Arogaya Yojana (PMJAY) and similar schemes failed to reduce CHE, largely due to double-billing and poor enforcement of price caps in private facilities. These issues persisted across multiple evaluations (Garg et al. 2019, 2020, 2022a, 2022b). In Mexico, the impact of Seguro Popular depended heavily on health service availability; CHE was significantly reduced in urban areas and well-resourced rural settings (Sosa-Rubí et al. 2011, Knaul et al. 2018), but no effect was found in underserved regions (Grogger et al. 2015). While Seguro Popular initially improved equity in OOPE and had a redistributive effect from 2003 to 2010, as service coverage expanded, its vertical equity impact declined (Garcia-Diaz et al. 2023).

Studies reporting on VHI, including CBHI, Long-Term Care Insurance, private VHI, and employer-sponsored schemes, differed substantially in target populations, premium structures, and benefit packages. Approximately one-third of the schemes targeted vulnerable groups, such as the disabled (Ma and Xu 2023), women's self-help groups (Raza et al. 2016), the poor (Aggarwal 2010, Thornton et al. 2010, Savitha and Kiran 2015a, Bousmah et al. 2022), garment workers (Ahmed et al. 2020), and schoolchildren (Jowett et al. 2003). Outcomes varied by scheme and context. For example, CBHI in Lao People's Democratic Republic (PDR) and Senegal showed gaps in financial protection because people often sought care outside the schemes due to exclusions, provider preferences, and medicine shortages. Similarly, the Yeshasvini programme in India increased private hospital use among insured individuals compared to uninsured and reduced borrowing for surgery, but led to increased borrowing for non-surgical inpatient care among better-off households, likely due to reliance on private facilities for treatments not covered by the scheme (Aggarwal 2010).

Studies also looked at actions taken to reform or improve insurance schemes, including the integration of fragmented programmes and the expansion of benefit coverage. The experience of the National Health Insurance scheme in Lao PDR, which saw the integration of CBHI, showed some improvements in equity. Income-based disparities in CHE were reduced, and healthcare utilization increased, especially for low-income households (Bodhisane and Pongpanich 2019, Bodhisane and Pongpanich 2022). However, patients from districts further from provincial hospitals had 3.8 times higher odds of CHE than those residing nearby, the poorest households were 200 times more likely to face CHE compared to the wealthiest, and households with chronic conditions were 108 times more likely to incur CHE than those without chronic conditions. The introduction of a 25% co-payment for certain expenses contributed to this increased financial burden on the poorest and sickest. Similarly, in China, integration of insurance schemes into URRBMI led to greater inequity for poorer and rural patients (based on changes to the concentration index), although inequity relating to the intensity of CHE and impoverishment among the poorest decreased (Wang et al. 2020) and effects on OOPE, CHE, and impoverishment were mixed (Wang et al. 2020, Zhou et al. 2022).

The expansion of the benefit package within the context of health insurance was used as a reform to improve financial protection in India [inclusion of multidrug-resistant TB (MDR-TB)], Kenya (inclusion of non-communicable disease services), and China (various reforms to increase inpatient and outpatient services included in the various insurance schemes’ benefit packages). China also relied on improving the level of compensation of the insured population through each of its health insurance schemes. Reforms that broadened coverage (e.g. Catastrophic Medical Insurance, Serious Illness Insurance, expanded NCMS) led to increased use of both inpatient and outpatient services, including institutional deliveries and postnatal care (You et al. 2016, Jiang et al. 2019a, Cao et al. 2022). Coverage of high-cost conditions (e.g. critical illness, MDR-TB) helped reduce delays in care and increased access to higher-quality facilities (Kundu et al. 2018, Yu et al. 2021).

Expanding benefit packages, e.g. by broadening coverage or increasing coverage of high-cost conditions, typically increased utilization, and in China, both the expanded NCMS and introduction of the Serious Illness Medical Insurance Scheme reduced impoverishment (Li et al. 2015, 2020). However, in several cases, it also perpetuated inequities and created new unintended vulnerabilities. For example, Critical Illness Insurance in China reduced CHE for the poorest but increased it for middle-income groups (Jiang et al. 2019a). In Shanghai, when insurance was expanded to cover heart stents, new cost-sharing rules accompanying the insurance required hospitals to shoulder part of the expense. In response, providers increased stent installations for patients with lower insurance coverage (where more of the cost fell on the patient) and reduced it among patients with generous coverage, where hospitals bore a larger share, indicating that provider behaviours shifted to mitigate the effects of cost sharing (Yuan et al. 2014).

#### Less studied interventions

Relatively few studies examined behaviour change interventions (*n* = 1), upgraded health facilities (*n* = 1), and social protection measures (*n* = 4). Social protection programmes involving cash transfers in Mexico, China, and Zambia showed some positive effects on reducing financial barriers to service use (Riumallo-Herl and Aguila 2019, Li et al. 2023b, Wang et al. 2023, Mori et al. 2024), but did not impact OOPE or CHE, and studies did not assess effects on impoverishment.

The only behaviour change intervention identified was Kangaroo Mother Care in India, which showed some positive effects on OOPE and reduced risk of impoverishment (Choudhary et al. 2022). One study from Nigeria assessed upgraded facilities implemented alongside a VHI scheme (Bonfrer et al. 2018). It found increased use of formal care (Bonfrer et al. 2018). However, the study also reported a reduction in healthcare spending among the uninsured, likely reflecting avoidance of the now more expensive services. This unintended effect is noteworthy given that 67% of the population did not enrol in the insurance scheme. In other contexts, upgraded facilities were introduced in tandem with other interventions, like insurance schemes or user fee reforms (Bauhoff et al. 2011, Zoidze et al. 2013, Gotsadze et al. 2015a, 2015b, Neelsen and O’Donnell 2017, Bonfrer et al. 2018, Okunogbe et al. 2022, Huang et al. 2023), but their effects were not separately evaluated, making it difficult to isolate their specific impact on financial protection outcomes.

#### Complex reforms

Complex reforms, consisting of multi-pronged or multiple interventions that could not be assigned to a single category, were studied in three countries: China, Cambodia, and Iran.

In China, three of the key programmes, the Targeted Poverty Alleviation programme, Health Poverty Alleviation Programme (HPAP), and Maternal and Child Health Policies and Programs for Poverty Alleviation were interrelated and designed as part of a broader anti-poverty strategy. In particular, HPAP is a multi-pronged initiative that aimed to alleviate multidimensional poverty through a cross-cutting approach of enhanced medical insurance, increased medical and charitable assistance, dynamic data-driven management, equitable resource distribution, strengthened healthcare infrastructure and human resources in underserved rural areas, and improved public health services (Li et al. 2023a). Evaluations of the HPAP and related anti-poverty reforms in China showed positive impacts (reduced CHE and impoverishment alongside increased service use) across studies (Chen and Pan 2019, Huang et al. 2023, Li et al. 2023a, Tang et al. 2023). Strong managerial accountability and interdepartmental coordination are highlighted as contributors to the success of HPAP (Chen and Pan 2019).

In contrast, China's New Health Care Reform (Huang et al. 2018, Xu et al. 2018, 2019, Liu et al. 2021, Tang et al. 2023, Cui et al. 2024) and studied reforms in Cambodia (Ensor et al. 2017) and Iran (Esmaeili et al. 2021) were more narrowly focused within the health sector and did not consistently improve financial protection outcomes. In Iran, one study examining the Health Transformation Plan alongside the Targeted Subsidies Plan, which replaced energy and food subsidies with targeted social assistance, found increases in CHE, which the authors attributed to inflationary pressures following implementation (Mohammadzadeh et al. 2023).

Across settings, contextual factors such as economic conditions, policy design, targeting mechanisms, and local health system capacity were cited as key to the success of complex interventions. For example, Iran's Health Transformation Plan showed limited or negative effects, partly due to macroeconomic constraints during implementation. Meanwhile, in China, a supportive political environment, strong government commitment to addressing poverty, and the availability of domestic funding created favourable conditions for the implementation of the Poverty Alleviation Programmes (Huang et al. 2023).

### Cost implications and financial sustainability of interventions

Intervention design affected the overall costs of providing care. For example, Vietnam's Health Care Fund for the Poor (Axelson et al. 2009), CBHI in India (Aggarwal 2010), China's universal medical insurance system (Xiong et al. 2018), and PFHI implementation in Georgia (Zoidze et al. 2013) and India (Parmar et al. 2023) led to increased use of costlier, higher-level or private care. In contrast, expanding outpatient coverage under China's New Cooperative Medical Scheme (NCMS) and increasing reimbursement for outpatient hypertension care both shifted care to lower-cost settings and reduced overall expenditures (Babiarz et al. 2010, Miao et al. 2018). Restructuring benefits to favour lower-cost, appropriate care may offer both financial risk protection and system efficiency, but such strategies remain under-evaluated. Strategic purchasing can improve cost efficiency, as seen in India's CBHI scheme, where negotiated tariffs were 40%–50% lower than private hospital rates (Aggarwal 2010).

SHI schemes can require substantial subsidies; for instance, employer-sponsored insurance for garment workers in Bangladesh costs US$193 annually, with employers contributing only US$6.3 per worker (Ahmed et al. 2020). Meanwhile, some interventions showed positive value-for-money by reducing household spending more than public expenditure on the intervention. In Benin and Burkina Faso, public spending on each case of delivery and caesarean fee exemptions was lower than the financial gain to households, indicating a net benefit (Witter et al. 2016). This likely reflected the way payments were structured, with health facilities delivering care without fully recovering costs. If facilities can maintain service quality without shifting costs to patients, such policies may generate efficiency gains at the system level. Similarly, India's Aarogyasri PFHI programme achieved reductions in household spending that exceeded programme costs by Rs. 40–60 per person per year, suggesting a positive return on public investment (Fan et al. 2012).

Provider payment reforms often have an explicit focus on overall cost reduction or containment, and in some cases, this result is reported to have been achieved. For example, analysis of the switch from fee-for-service to episode-based payments in China found a net decrease in government expenditure of just over 60% (*P* < 0.01) (Meng et al. 2019b). However, some results are more complex. Provider payment reforms were indicated by several studies as contributing to supplier-induced demand. This is discussed in greater detail in the intervention-specific analyses below. Such behaviour was also observed following a medicine coverage policy in China (Chen et al. 2017).

## Discussion

Financial protection in healthcare is influenced by individual, household and social factors, local contexts, illness characteristics, health expenditure types and costs, service quality and accessibility, and broader health system structures, including financing and regulation. Effective interventions to enhance financial protection can target any of these dimensions and be categorized in multiple ways. This review aimed to synthesize evidence on the effects of various interventions on improving financial protection related to healthcare, analysing it by several sub-themes. Based on the effects of interventions across categories, we identify below some cross-cutting messages that help explain variation in outcomes and offer policy-relevant insights for designing and implementing more equitable financial protection strategies.

### Reaching target groups

Whilst some targeted interventions achieved stronger financial protection, this was not consistently the case. In fact, some targeted interventions demonstrated consistently poor outcomes across multiple studies (Shi et al. 2010, Liu et al. 2017, Chen et al. 2023a). The Medical Financial Assistance programme in China, which specifically targeted low-income households, did not demonstrate significant impact on any financial protection outcomes.

It was unclear whether poor outcomes in targeted interventions were due to poor design, weak targeting, underlying inequities, or implementation challenges. Where interventions targeted the poor, e.g. for demand-side financing schemes (Senegal and Bangladesh), targeted exemptions (Burkina Faso) or for the purpose of calculating insurance premiums (Ghana), this is often challenging and costly from an administrative perspective, leading to ineffective targeting (Nguyen et al. 2011, Nguyen et al. 2012a, 2012b, Ridde et al. 2015, Bousmah et al. 2022). PhilHealth reimbursements under the sponsored programme in the Philippines disproportionately benefited wealthier patients, leaving poorer populations under-protected due to targeting inefficiencies.

However, the evidence also indicates that when targeting is done effectively, it can have beneficial equity impacts. For example, Mexico's Seguro Popular showed beneficial equity effects when it targeted the poor, but these diminished as coverage expanded to richer households, reducing the targeting focus and leading to total resources being spread too thin (Zhu et al. 2022, Garcia-Diaz et al. 2023). When targeting is not a core design feature, interventions may inadvertently worsen inequities. This was evident in Iran and Türkiye's family medicine programmes, which resulted in poorer equity outcomes for lower-income groups (Homaie Rad et al. 2017, Erus 2020).

### Implementation context

The broader context within which interventions are implemented, including macroeconomic conditions, geography, and sociocultural diversity, can affect intervention impact. For example, in Türkiye, the growth of the economy after 2000 positively influenced the success of the health financing reforms (Yardim et al. 2014). By contrast, in Iran, the Health Transformation Plan and the Urban Family Physician programme were introduced during a period of economic instability, inflation, sanctions, and declining oil revenues, which constrained public funding and undermined outcomes (Homaie Rad et al. 2017, Ahmadnezhad et al. 2019, Darvishi et al. 2021, Malekroudi et al. 2023). Contextual factors also affect implementation and intervention fidelity. In Yunnan province, China, the Urban Employee Basic Medical Insurance (UEMBI) scheme faced implementation challenges due to mountainous terrain, cultural diversity, and limited service delivery capacity (Huang et al. 2023). In Bangladesh, cultural preferences for informal providers limited uptake of a CBHI programme (Ahmed et al. 2020).

The success of financial protection policies also depends heavily on the health system context. A recent study found that reducing the financial burden of healthcare relies on multiple system attributes, such as revenue generation, pooling, or purchasing, being strong and synergistically aligned. In other words, building a health financing system that ensures financial protection requires coordinated design across several dimensions, not isolated fixes (Hsu et al. 2025).

Supply-side barriers limit the effectiveness of financial protection interventions across categories. Factors like insufficient healthcare infrastructure, lack of supplies, drugs and equipment, low availability, and quality of the health workforce are mentioned across multiple settings as challenges to intervention success [e.g. China (Dai et al. 2016, Ma and Xu 2023, Zhu et al. 2024), Vietnam, Rwanda, Lao PDR (Bodhisane and Pongpanich 2022), India (Ahmed and Mahapatro 2023, Parmar et al. 2023), Mexico, Madagascar, Nepal (Sunny et al. 2021), Sierra Leone (Edoka et al. 2016), Zambia (Masiye et al. 2016), West Africa (Witter et al. 2016), Tanzania]. In voluntary insurance schemes, supply-side factors limit enrolment, highlighting the need for broader health system reforms and supply-side support for interventions (Nguyen et al. 2012b).

Studies highlight that interventions can worsen supply-side challenges by enabling unethical supplier practices such as supplier-induced demand, up-charging, and double-billing. For example, in Iran, supplier-induced demand was linked to systemic issues, such as inefficient physician contracts, weak referral systems, fragmented governance, poor regulation, and health market problems like information asymmetry and clinical uncertainty (Seyedin et al. 2020). These factors are supported by a scoping review, which notes that healthcare demand is shaped by both supply-side factors (e.g. physician income incentives, staffing ratios, tariffs, and hospital type) and demand-side factors (e.g. patient characteristics and insurance coverage) (Mohammadshahi et al. 2019).

Among included studies, lack of awareness of insurance benefits is a commonly mentioned demand-side factor limiting intervention effects [Nicaragua/Seguro Facultativo de Salud (Bauhoff et al. 2011), China/Long-Term Care Insurance (Ma and Xu 2023), India/PFHI (Garg et al. 2019), Georgia/PFHI (Gotsadze et al. 2015b), West Africa/exemptions (Witter et al. 2016), Madagascar/health equity fund (Honda and Hanson 2013), Senegal/cash transfers (Bousmah et al. 2022)]. Similarly, costs of transport, food and other indirect costs create barriers to healthcare and impact on level of financial protection, even once financial protection for direct costs is improved (Rwanda, Senegal—Koch et al. 2022, Ly et al. 2022). The experience of China's NEMS reform highlights the importance of understanding how components such as drug costs, service pricing, and consumables interact during large-scale health system changes (Chen et al. 2020).

### Complex challenges require multi-pronged solutions

Financial protection in health is a complex challenge that demands multi-pronged, context-sensitive solutions. The evidence clearly shows that no single intervention type is sufficient. Instead, efforts to improve financial protection must address interconnected factors both within and beyond the health sector. Pre-emptive attention to how systemic features shape implementation and outcomes is important, along with tools to support navigation of the political economy of policy adoption (World Health Organization 2024).

China's HPAP illustrates how favourable political and financial conditions can enable ambitious, multi-sectoral reforms. Its success suggests that, while targeting vulnerable populations with health-related interventions (e.g. free care) is often assumed to be equitable, in practice, broader structural factors shape financial protection. Interventions that address these systemic factors and inequities are better placed to improve outcomes than those focused solely on targeting through the health system.

A policy analysis on measuring financial protection also reinforces the need for a multifaceted approach (Wagstaff 2008). The analysis found that effects of insurance interventions varied, especially when limited benefits, high cost-sharing, or inadequate provider payment led to increased OOPE or informal payments. In some contexts, supply-side measures like treatment protocols and drug lists had proven more effective. Multiple policy instruments used in coordination are needed to achieve financial protection goals.

### Gaps in the evidence

Understudied areas included interventions targeting revenue mobilization or resource allocation, governance-related interventions, such as those involving provider autonomy or regulation of the private sector, and social determinants of health. Notable gaps in the literature relate to the assessment of interventions targeting revenue mobilization and resource allocation, governance arrangements, and social determinants of health. These areas remain underexplored in terms of their impact on financial protection outcomes. However, in some cases, studied interventions may have indirectly involved such components. For instance, no interventions were classified under ‘revenue mobilization’ or ‘resource allocation’ although many studies assessed demand-side financing approaches that likely implied changes in these functions.

While several studies addressed medicine-related interventions, none focused on what could be impactful areas of strengthening supply chains, making procurement more efficient and promoting rational prescribing. Social protection was also rarely studied, despite its potential relevance.

In several cases, interventions which are likely to impact financial protection were studied for their effect on health outcomes, quality of care, or service utilization and access, but not financial protection outcomes. This gap is particularly apparent in the literature on performance-based financing (Bonfrer et al. 2013, Falisse et al. 2015, Diaconu et al. 2021) and underscores the lack of focus on financial protection as an outcome. However, it is important to note that a lack of evidence does not necessarily mean a lack of impact.

Where equity was assessed, interventions were more likely to reveal harmful effects. Broad reforms that did not specifically target vulnerable groups sometimes produced regressive outcomes, disproportionately benefiting wealthier populations. They also risked overstretching limited public resources. For example, Mexico's Seguro Popular initially improved equity, but its impact declined as coverage expanded to wealthier households, diluting the focus on the poor and overstretching resources (Garcia-Diaz et al. 2023). In Zambia, removing user fees improved access overall, but also led wealthier individuals to shift from private to public facilities, potentially impeding poorer users’ access (Lépine et al. 2018). Even targeted interventions faced challenges, often excluding marginalized groups due to systemic inequities like income, education, gender, and geography. Uganda's zero-interest loan programme failed to reach the poorest individuals outside community groups (Nannini et al. 2021). In India, incentives for institutional deliveries were less likely to be taken up by older women and those living farther from hospitals (Gopalan and Durairaj 2012), while a maternity care cash transfer was regressive, leading poorer households to spend a higher share of their disposable income than richer households (Mukherjee and Singh 2018). Informal payments for the poor (those with low social capital) were also an issue (Mukherjee and Singh 2018). In contrast, HPAP in China showed that a programme that focused on addressing structural barriers to equitable financial protection (e.g. the programme championed the equitable distribution of medical resources across urban and rural landscapes, addressed public health priorities, as well as social protections), consistently improved financial protection and equity outcomes (Chen and Pan 2019, Li et al. 2023a).

Evidence on overall costs of service provision and cost-effectiveness was limited and varied in quality. Intervention design played a significant role in shaping costs: some increased spending, by promoting use of higher-level or private care, or due to compensatory behaviour by providers affected by cost-containment reforms, while others reduced expenditures by shifting care to lower-cost settings. Restructuring benefits to prioritize appropriate, lower-cost care may improve both financial protection and efficiency, but this remains under-evaluated.

### Strengths and limitations

This review synthesizes published literature on the effects of interventions on financial protection, covering a broader evidence base than previous studies. By identifying studies based on outcomes rather than intervention type, it offers a systematic and balanced view of what has and has not been studied for its effect on financial protection. This inclusive approach revealed evidence gaps that had not been noted elsewhere. Consistency with prior reviews (e.g. Jalali et al. 2021, Bhatia et al. 2022, Eze et al. 2023, Sugunan et al. 2023) reinforces research reliability, strengthening confidence in key conclusions and providing a fuller understanding of financial protection strategies that have been studied.

Several limitations should be noted when interpreting this review's findings. We found substantial variation in how financial protection outcomes are defined, measured and reported, including differences in data sources, recall periods, population scope, measurement of exposure, and metrics used, thus influencing how results are interpreted and contributing to the heterogeneity in reported effects. OOPE was the most commonly assessed outcome, with impoverishment being the least assessed. However, the meaning of OOPE varies depending on measurement and context. OOPE alone does not capture the true state of financial protection unless interpreted within its specific context. Reductions in household OOPE may signal foregone care rather than improved protection, while increases could reflect better access due to higher service use, not necessarily greater financial hardship. Measures like OOPE as a percentage of total health expenditure at the country level can be more informative. In this review, we reported OOPE as presented without further analysing how it interacted with other financial protection measures in specific contexts.

Outcome measurement varies widely across studies, complicating comparisons. For example, in Cambodia, patient diaries showed increased outpatient use after fee exemptions, while household surveys showed no change (Korachais et al. 2019). In China, NCMS impacts varied by statistical model (Babiarz et al. 2010). Such inconsistencies highlight the limitations of categorizing effects simply as beneficial, harmful, or neutral, and the importance of context in interpreting results. The included studies varied widely in design; 70% were observational, 25% quasi-experimental, and only 5% experimental. Common methodological issues included reliance on self-reporting, small sample sizes, inadequate control for confounders, and weak intervention fidelity, all of which affect reliability and generalizability. Intervention diversity also complicated analysis, as many studies combined components, making categorization difficult. To address this, we created a ‘complex reform’ category and noted cross-cutting elements during analysis. Generalizability was further limited by studies focusing on specific population groups or sub-national contexts. Broader effects on untargeted populations were often unmeasured or unreported. Only empirical, published literature was included; thus, this review was limited in its ability to assess intervention design and implementation, including political, economic, or bureaucratic factors that may have impacted the effectiveness of interventions. Additionally, the evidence base itself is biased by what has been studied and published. Future research should incorporate grey literature, potentially in the context of in-depth country case studies following policy impacts over time, also triangulating findings with national health coverage data. Disaggregating results by intervention mechanisms and effects on sub-populations would also enhance the relevance of findings for policy. We underscore the need for more systematic analysis of financial protection outcomes beyond health insurance and for more consistent reporting of costs and implementation challenges.

## Conclusion

This study finds that there is no single measure that consistently improves health-related financial protection for all. Rather, a range of policies, if well designed and targeted in their contexts, can have beneficial effects. Improving equity in financial protection involves reducing direct financial barriers to care as well as tackling systemic inequalities that drive up care costs or increase vulnerability to financial hardship. Interventions should consider the full range of factors that contribute to financial hardship, including indirect costs, access barriers, and structural inequities to ensure they are both effective and equitable. Finally, broader contextual factors, such as governance, administrative capacity, health system readiness, and alignment with social protection systems, also shape whether interventions reach those most in need and achieve their intended effects. Continuous monitoring and adaptation of interventions is important for identifying unintended consequences and adapting to ensure interventions are effective and equitable. Financial protection goals should be explicit and prioritized, and financial protection outcomes should be monitored for a wider range of policy interventions than has hitherto been the case.

## Supplementary Material

czag065_Supplementary_Data

## Data Availability

No new data were generated or analysed in support of this research.

## High income authors

The work was commissioned to generate insights on how to reduce financial hardship from healthcare spending. It had a global focus and did not specify any particular region, so inclusion of specific regional experts in the review group was not required.

## Reflexivity statement

This review was undertaken by authors based in high-income countries, all female, with extensive experience of working on financial barriers and financial protection in healthcare over many decades in low- and middle-income countries. We benefited from the wisdom of our advisory group, which included leading global experts in the field from multiple regions.
