# Supplementary material for: Assessing intervention effects on healthcare-related financial protection in low- and middle-income countries: a rapid evidence review
Source: Health Policy Plan. 2026 May 12;41(7):1307–33. doi: 10.1093/heapol/czag065 (PMC13481695; doi:10.1093/heapol/czag065)
Supplement: Supplementary file 1 [file czag065_supplementary_data.zip › Completed PRISMA_2020_checklist.docx]

| **Section and Topic** | **Item #** | **Checklist item** | **Location where item is reported** |
| --- | --- | --- | --- |
| **TITLE** | | |  |
| Title | 1 | Identify the report as a systematic review. | Identified as evidence review (not systematic review) in title (pg 1) |
| **ABSTRACT** | | |  |
| Abstract | 2 | See the PRISMA 2020 for Abstracts checklist. | See below this table |
| **INTRODUCTION** | | |  |
| Rationale | 3 | Describe the rationale for the review in the context of existing knowledge. | Clear guidelines and policy recommendations are needed to ensure governments are able to prioritise financial protection in their reform agenda. However, which interventions work for financial protection is not clear from the current literature.  We summarize that the existing evidence reviews address some specific topics but do not investigate the full range of interventions which could address financial protection. |
| Objectives | 4 | Provide an explicit statement of the objective(s) or question(s) the review addresses. | Top of pg 4 |
| **METHODS** | | |  |
| Eligibility criteria | 5 | Specify the inclusion and exclusion criteria for the review and how studies were grouped for the syntheses. | Pg 4-5 and Table 1 |
| Information sources | 6 | Specify all databases, registers, websites, organisations, reference lists and other sources searched or consulted to identify studies. Specify the date when each source was last searched or consulted. | Pg 4 (line 105) |
| Search strategy | 7 | Present the full search strategies for all databases, registers and websites, including any filters and limits used. | The terms searched are presented in Table 1. Page 4 specifies that there were no filters or limits applied. |
| Selection process | 8 | Specify the methods used to decide whether a study met the inclusion criteria of the review, including how many reviewers screened each record and each report retrieved, whether they worked independently, and if applicable, details of automation tools used in the process. | Pg 4 (line 109-111) |
| Data collection process | 9 | Specify the methods used to collect data from reports, including how many reviewers collected data from each report, whether they worked independently, any processes for obtaining or confirming data from study investigators, and if applicable, details of automation tools used in the process. | Pg 5 (line 118 to 125) |
| Data items | 10a | List and define all outcomes for which data were sought. Specify whether all results that were compatible with each outcome domain in each study were sought (e.g. for all measures, time points, analyses), and if not, the methods used to decide which results to collect. | Pg 5 (line 119-124, line 126 to 135) with further details on the data within each outcome domain presented in table 7 of the results |
|  | 10b | List and define all other variables for which data were sought (e.g. participant and intervention characteristics, funding sources). Describe any assumptions made about any missing or unclear information. | Pg 5 (line 119-124, line 126 to 135) with further details on the data within each outcome domain presented in table 7 of the results |
| Study risk of bias assessment | 11 | Specify the methods used to assess risk of bias in the included studies, including details of the tool(s) used, how many reviewers assessed each study and whether they worked independently, and if applicable, details of automation tools used in the process. | Pg 5 (line 136-141) |
| Effect measures | 12 | Specify for each outcome the effect measure(s) (e.g. risk ratio, mean difference) used in the synthesis or presentation of results. | Pg 5 and Table 7 of the results |
| Synthesis methods | 13a | Describe the processes used to decide which studies were eligible for each synthesis (e.g. tabulating the study intervention characteristics and comparing against the planned groups for each synthesis (item #5)). | Pg 5 (line 126 to 132) |
|  | 13b | Describe any methods required to prepare the data for presentation or synthesis, such as handling of missing summary statistics, or data conversions. | Not applicable since narrative synthesis methods were used (pg 5, line 142-145) |
|  | 13c | Describe any methods used to tabulate or visually display results of individual studies and syntheses. | Pg 5 (Line 129 to 135) and pg 6 (line 162 to 175) |
|  | 13d | Describe any methods used to synthesize results and provide a rationale for the choice(s). If meta-analysis was performed, describe the model(s), method(s) to identify the presence and extent of statistical heterogeneity, and software package(s) used. | Pg 5 (line 142-145) |
|  | 13e | Describe any methods used to explore possible causes of heterogeneity among study results (e.g. subgroup analysis, meta-regression). | Not applicable, heterogeneity was addressed through narrative synthesis. |
|  | 13f | Describe any sensitivity analyses conducted to assess robustness of the synthesized results. | Not applicable. |
| Reporting bias assessment | 14 | Describe any methods used to assess risk of bias due to missing results in a synthesis (arising from reporting biases). | Not applicable. |
| Certainty assessment | 15 | Describe any methods used to assess certainty (or confidence) in the body of evidence for an outcome. | Not applicable. |
| **RESULTS** | | |  |
| Study selection | 16a | Describe the results of the search and selection process, from the number of records identified in the search to the number of studies included in the review, ideally using a flow diagram. | PRISMA diagram (Figure 6) |
|  | 16b | Cite studies that might appear to meet the inclusion criteria, but which were excluded, and explain why they were excluded. | This was not done due to a need to limit what is included to meet word count restrictions. Reasons for exclusion are presented in Figure 6. |
| Study characteristics | 17 | Cite each included study and present its characteristics. | Annex 1 |
| Risk of bias in studies | 18 | Present assessments of risk of bias for each included study. | This is not included given that this is not a systematic review. Studies which were not excluded due to weak methodological quality were given equal weight and their collective inclusion was guided by the principle of triangulation (see page 5). |
| Results of individual studies | 19 | For all outcomes, present, for each study: (a) summary statistics for each group (where appropriate) and (b) an effect estimate and its precision (e.g. confidence/credible interval), ideally using structured tables or plots. | Summaries of outcomes are presented in figures 1 to 5 (see page 8) |
| Results of syntheses | 20a | For each synthesis, briefly summarise the characteristics and risk of bias among contributing studies. | The study characteristics are presented in the results for each analysis category. Risk of bias is not reported by study, as set out above (point 18 – Risk of bias in studies). |
|  | 20b | Present results of all statistical syntheses conducted. If meta-analysis was done, present for each the summary estimate and its precision (e.g. confidence/credible interval) and measures of statistical heterogeneity. If comparing groups, describe the direction of the effect. | Not applicable – a narrative synthesis approach was used. |
|  | 20c | Present results of all investigations of possible causes of heterogeneity among study results. | These are narratively described throughout the results. |
|  | 20d | Present results of all sensitivity analyses conducted to assess the robustness of the synthesized results. | Not applicable. |
| Reporting biases | 21 | Present assessments of risk of bias due to missing results (arising from reporting biases) for each synthesis assessed. | Not applicable. |
| Certainty of evidence | 22 | Present assessments of certainty (or confidence) in the body of evidence for each outcome assessed. | Not applicable. |
| **DISCUSSION** | | |  |
| Discussion | 23a | Provide a general interpretation of the results in the context of other evidence. | Pg 17 to 19 |
|  | 23b | Discuss any limitations of the evidence included in the review. | Pg 22 |
|  | 23c | Discuss any limitations of the review processes used. | Pg 22 |
|  | 23d | Discuss implications of the results for practice, policy, and future research. | Pg 19-20 and pg 22-23 |
| **OTHER INFORMATION** | | |  |
| Registration and protocol | 24a | Provide registration information for the review, including register name and registration number, or state that the review was not registered. | The review was not registered. |
|  | 24b | Indicate where the review protocol can be accessed, or state that a protocol was not prepared. | A protocol was not published; a protocol was submitted to FCDO (the funder) when this research commenced and is available upon request from the authors. |
|  | 24c | Describe and explain any amendments to information provided at registration or in the protocol. | Not applicable. |
| Support | 25 | Describe sources of financial or non-financial support for the review, and the role of the funders or sponsors in the review. | Title page. |
| Competing interests | 26 | Declare any competing interests of review authors. | Title page: the authors have no competing interests to declare. |
| Availability of data, code and other materials | 27 | Report which of the following are publicly available and where they can be found: template data collection forms; data extracted from included studies; data used for all analyses; analytic code; any other materials used in the review. | The data used for analyses and data collection forms are not publicly available. Data extracted from included studies is partly available in Annex 1, the table of included studies (key study characteristics) and is available in the full FCDO report (key outcomes analysed within each category of intervention) |

*From:*  Page MJ, McKenzie JE, Bossuyt PM, Boutron I, Hoffmann TC, Mulrow CD, et al. The PRISMA 2020 statement: an updated guideline for reporting systematic reviews. BMJ 2021;372:n71. doi: 10.1136/bmj.n71

**Abstract checklist**

| **Section and Topic** | **Item #** | **Checklist item** | **Reported (Yes/No)** |
| --- | --- | --- | --- |
| **TITLE** | | |  |
| Title | 1 | Identify the report as a systematic review. | Yes as evidence review |
| **BACKGROUND** | | |  |
| Objectives | 2 | Provide an explicit statement of the main objective(s) or question(s) the review addresses. | Yes |
| **METHODS** | | |  |
| Eligibility criteria | 3 | Specify the inclusion and exclusion criteria for the review. | Yes |
| Information sources | 4 | Specify the information sources (e.g. databases, registers) used to identify studies and the date when each was last searched. | Not in abstract |
| Risk of bias | 5 | Specify the methods used to assess risk of bias in the included studies. | Not in abstract |
| Synthesis of results | 6 | Specify the methods used to present and synthesise results. | Yes |
| **RESULTS** | | |  |
| Included studies | 7 | Give the total number of included studies and participants and summarise relevant characteristics of studies. | Yes |
| Synthesis of results | 8 | Present results for main outcomes, preferably indicating the number of included studies and participants for each. If meta-analysis was done, report the summary estimate and confidence/credible interval. If comparing groups, indicate the direction of the effect (i.e. which group is favoured). | Yes |
| **DISCUSSION** | | |  |
| Limitations of evidence | 9 | Provide a brief summary of the limitations of the evidence included in the review (e.g. study risk of bias, inconsistency and imprecision). | Not in abstract |
| Interpretation | 10 | Provide a general interpretation of the results and important implications. | Yes |
| **OTHER** | | |  |
| Funding | 11 | Specify the primary source of funding for the review. | In title page, not in abstract |
| Registration | 12 | Provide the register name and registration number. | Not applicable |

*From:*  Page MJ, McKenzie JE, Bossuyt PM, Boutron I, Hoffmann TC, Mulrow CD, et al. The PRISMA 2020 statement: an updated guideline for reporting systematic reviews. BMJ 2021;372:n71. doi: 10.1136/bmj.n71
