# Supplementary material for: Assessing intervention effects on healthcare-related financial protection in low- and middle-income countries: a rapid evidence review
Source: Health Policy Plan. 2026 May 12;41(7):1307–33. doi: 10.1093/heapol/czag065 (PMC13481695; doi:10.1093/heapol/czag065)
Supplement: Supplementary file 1 [file czag065_supplementary_data.zip › Supplementary - Complete Annex 1 - unlinked citations.docx]

# Annex 1. Overview of included studies

| Citation | Number of records | Studied categories | Intervention overview | Country | Study aim | Studied outcomes |
| --- | --- | --- | --- | --- | --- | --- |
| (Aashima and Sharma, 2024) | 3 | - Insurance integration/ overarching assessment of multiple schemes - SHI - VHI | - Rashtriya Swasthya Bima Yojana (RSBY) (government health insurance programme launched in 2008 providing hospital-based healthcare services to families living below the poverty line) - Pradhan Mantri Jan Arogaya Yojana (PMJAY) (PFHI launched in 2018, replacing RSBY) - Sampoorna Suraksha Programme CBHI | India | Examined the impact of several health insurance schemes on inpatient care use. | OOPE, CHE, Impoverishment, Service utilisation |
| (Abrokwah et al., 2014) | 1 | - SHI | - National Health Insurance Scheme | Ghana | Studied how the national health insurance scheme affected prenatal care use and expenditures, utilising district-level rollout as exogenous variation. | OOPE, CHE, Equity |
| (Aggarwal, 2010) | 1 | - VHI | - Yeshasvini (CBHI) | India | Evaluated the health and economic impacts of the Yeshasvini community-based health insurance programme for the poor on health and economic outcomes. | OOPE, Service utilisation |
| (Ahmadnezhad et al., 2019) | 1 | - Complex | - Health Transformation Plan (also referred to as Health Sector Evolution Plan), implemented in three phases   Phase 1 (2014): Reduced out-of-pocket payments, strengthened hospital care, improved quality, and expanded financial protection.  Phase 2 (2014): Expanded primary healthcare and implemented the family physician programme.  Phase 3 (2015–): Reformed medical tariffs, controlled informal payments, and revised provider payment systems. | Iran | Investigated the impact of the Health Transformation Plan on the level and pattern of OOPE and CHE. | OOPE |
| (Ahmadnezhad et al., 2023) | 1 | - Complex | - Health Transformation Plan (also referred to as Health Sector Evolution Plan) | Iran | Investigated impoverishment due to OOPE and the impact on poverty pre- and post-Health Transformation Plan. | OOPE |
| (Ahmed and Mahapatro, 2023) | 1 | - Insurance integration/ overarching assessment of multiple schemes | - Various health insurance schemes | India | Analysed the effect of insurance coverage on OOPE and assessed subsidy distribution across income groups. | OOPE, CHE, Other |
| (Ahmed et al., 2020) | 1 | - VHI | - Employer-sponsored health insurance scheme | Bangladesh | Estimated the effect of an employer-sponsored health insurance scheme on healthcare utilisation of medically trained providers and reduction of OOPE among garment workers. | OOPE, CHE, Other |
| (Aji et al., 2013) | 2 | - SHI - PFHI | - Civil servant health insurance (Askes) - Employee health insurance (Jamsostek) - Health insurance for poor people (Askeskin) | Indonesia | Investigated the impact of each of Indonesia’s three largest insurance schemes on reducing OOPE. | OOPE, Equity |
| (Alkenbrack and Lindelow, 2015) | 1 | - VHI | - Community Based Health Insurance (CBHI) implemented by government alongside other health schemes to cover the informal sector | Lao PDR | Estimate the impact of CBHI CBHI for individuals working in the informal sector, who are not covered by formal social health insurance, on health service utilisation and OOPE. | OOPE, Equity |
| (Anselmi et al., 2017) | 1 | - Provider payment reforms | - Pay-for-performance scheme which rewards facilities with performance-based bonuses for maternal and child health targets, shared mainly with health workers. | Tanzania | Tested the causal pathways through which pay-for-performance schemes influence maternal care outcomes, focusing on institutional deliveries and uptake of antimalarial drugs during pregnancy. | CHE, Service utilisation, Equity |
| (Assari Arani et al., 2018) | 1 | - Complex | - Health Transformation Plan (also referred to as Health Sector Evolution Plan) | Iran | Evaluated the effects of two years of implementation of the Health Sector Evolution Plan on health equity indices. | OOPE, Service utilisation, Other |
| (Aryeetey et al., 2016) | 1 | - SHI | - National Health Insurance Scheme which involves voluntary enrolment for persons in the informal sector with an income-dependent premium. | Ghana | Analysed the effect of health insurance on household OOPE, CHE, and poverty. | OOPE, Service utilisation, Other |
| (Atella et al., 2015) | 1 | - SHI | - Urban Resident Basic Medical Insurance (URBMI) (covering non-employed urban residents, such as children, the disabled, and the poor, with shared premiums between individuals and the government) | China | Evaluated the impact of the 1998 Chinese health reform (Basic Insurance Scheme) on OOPE and savings. | Impoverishment, Service utilisation, Equity |
| (Axelson et al., 2009) | 1 | - SHI | Community Health Insurance covers inpatient treatment at state hospitals and outpatient services but only includes inpatient drugs on a Ministry of Health list, typically excluding outpatient medications. It  comprises three programs covering 41% of the population as of 2007:   - Social Health Insurance: Covers 9% of the formally employed, retirees, and dependents of military and police officers, funded by a payroll tax of up to 3%. - Health Care Fund for the Poor: Covers 18% of the population and receives three-quarters of its funding from the central government. - Free Health Care for Children Under 6: Covers 11% of children, funded entirely by the central government. | Vietnam | Evaluated the impact of the Health Care Fund for the Poor on healthcare utilisation and expenditures during its initial implementation period. | OOPE, CHE |
| (Aye et al., 2023) | 2 | - Provider payment reforms - User fee reforms | - Performance based financing implemented alongside gratuité policies (user fee removal) for pregnant and lactating women and children under 5 between 2014 and 2018 - Removal of user fees for delivery and children’s healthcare | Burkina Faso | Assessed the effect of gratuité and performance-based financing policies on facility-based OOPEs for outpatient services. | OOPE, CHE |
| (Babiarz et al., 2010) | 1 | - SHI | - New Cooperative Medical Scheme (NCMS) started in 2003, thereafter New Rural Cooperative Medical Scheme (NRCMS), started in 2007 | China | Determined whether the NCMS and its attributes affected the operation and use of village health clinics. | OOPE, CHE, Equity, Other |
| (Bauhoff et al., 2011) | 1 | - PFHI | - Government-funded medical insurance for the poor | Georgia | Evaluated the impact of medical insurance for the poor on utilisation, financial risk protection, and health behavior and management. | OOPE, CHE, Impoverishment, Service utilisation, Equity |
| (Binyaruka et al., 2015) | 1 | - Provider payment reforms | - Pay-for-performance scheme which rewards facilities with performance-based bonuses for maternal and child health targets, shared mainly with health workers. | Tanzania | Examined the effect of a government pay-for-performance scheme on the utilisation, quality, and user costs of health services. | OOPE, Service utilisation |
| (Bodhisane and Pongpanich, 2019) | 1 | - Insurance integration/ overarching assessment of multiple schemes | - Implementation of National Health Insurance through a combination of three healthcare schemes, offering outpatient and inpatient services, where the contribution rates depend on the location of the health service provide | Lao PDR | This study aims to assess the effectiveness of the National Health Insurance in terms of its accessibility and in providing financial protection from catastrophic health expenditure as compared with the existing CBHI scheme | OOPE, CHE, Equity, Other |
| (Bodhisane and Pongpanich, 2022) | 1 | - Insurance integration/ overarching assessment of multiple schemes | - Implementation of National Health Insurance through a combination of three healthcare schemes, offering outpatient and inpatient services, where the contribution rates depend on the location of the health service provide | Lao PDR | This study aims to analyse the effects of the Lao National Health Insurance on issues of accessibility and the possibility of encountering catastrophic health expenditures for patients with chronic kidney disease | OOPE, Equity |
| (Bonfrer et al., 2018) | 2 | - VHI - Upgraded health facilities | - Voluntary CBHI programme that subsidised the cost of annual premiums for enrollees and upgraded selected health facilities in the intervention areas. Enrolment in the insurance programme was at the individual level and could occur on a monthly basis. Coverage lasted for a full year, after which it needed to be renewed. Enrolment and renewal were voluntary. - Upgrading of two largest health facilities in the Afon and Aboto Oja districts in Central Kwara contracted by the health insurance scheme | Nigeria | Estimated the effects of the Kwara State Health Insurance programme (subsidized low-cost VHI and facility upgrades) on insured and uninsured populations in Nigeria. | OOPE, CHE, Service utilisation |
| (Bose and Dutta, 2018) | 1 | - Reducing cost and increasing access to essential medicine | - Free medicine scheme in all public hospitals in Tamil Nadu and Rajasthan - Fair Price Medicine Shop scheme, a public-private-partnership model that subsidised price of medicines in both public and private hospitals in West Bengal | India | Studied utilisation patterns of public inpatient care facilities, effectiveness of strategies to reduce OOPE, and equity in services in three Indian states. | OOPE, Equity |
| (Bousmah et al., 2021) | 1 | - User fee reforms | - Removal of user fees for antiretroviral treatment | Cameroon | Explored the effects of free antiretroviral treatment policy on CHE risk by socioeconomic status among people living with HIV (PLHIV) in Cameroon’s antiretroviral treatment access programme. | OOPE, Equity |
| (Bousmah et al., 2022) | 1 | - VHI | - Voluntary CBHI scheme that includes a general scheme (CBHI-1), which is partially subsidised, and a special scheme for the poor (CBHI-2), which is fully financed by the state (registration and free care). | Senegal | Assessed the effects of the Programme National de Bourses de Securite Familiale (PNBSF) on health service utilisation and financial protection in Senegal. | OOPE, Service utilisation, Equity |
| (Brugiavini and Pace, 2016) | 1 | - SHI | - National Health Insurance Scheme which involves voluntary enrolment for persons in the informal sector with an income-dependent premium. | Ghana | Investigated how national health insurance affected utilisation of maternal health services and OOPE | OOPE, Equity |
| (Caballes et al., 2012) | 3 | - SHI - PFHI - User fee reforms | - PhilHealth: National social health insurance scheme run by the Philippine Health Insurance Corporation, primarily funding in-hospital care - Sponsored Program (insurance premium subsidies for low-income individuals) - Hospital Provided Discounts | Philippines | Assessed the ability of PhilHealth vs. institutional discounts to offer financial protection for indigent hospitalized patients. | CHE, Service utilisation |
| (Cai et al., 2022) | 1 | - Reducing cost and increasing access to essential medicine | - National Drug Price Negotiation policy | China | Assessed the impact of this policy on the utilisation, cost, and accessibility of 17 anticancer medicines | CHE, Service utilisation, Equity |
| (Cao et al., 2022) | 1 | - Expanded benefit package or enhanced compensation | - Catastrophic Medical Insurance | China | Examined the effect of Catastrophic Medical Insurance on healthcare expenditures among the elderly (65+). | OOPE, Equity |
| (Celhay et al., 2019) | 1 | - PFHI | - Medical Insurance Century XXI (SMSXXI) programme, to provide insurance to children younger than 5 years without social security, implemented as part of Seguro Popular (voluntary national health insurance scheme to provide health care for the poor) | Mexico | Examined the effects of Seguro Popular, on health outcomes and financial protection for Mexico's children using multiple nationally representative surveys and administrative data sources spanning 2001-2006. | OOPE, Equity |
| (Chen and Ning, 2022) | 1 | - VHI | - Long Term Care Insurance | China | This study aims to examine the policy treatment effect of long-term care insurance (LTCI) on health care utilisation and out-of-pocket health expenditure (OOP) in China | OOPE, Impoverishment |
| (Chen and Pan, 2019) | 1 | - Complex | - Health Poverty Alleviation Programme (HPAP) is a multi-component poverty alleviation programme that strengthened health insurance and medical assistance for poor rural households, improved targeting through dynamic data systems, promoted equitable urban–rural resource distribution, upgraded healthcare infrastructure, expanded telemedicine, and incentivised the deployment of health workers to underserved areas. | China | Explored the impact of HPAP on financial risk protection in rural households. | OOPE, Impoverishment |
| (Chen et al., 2017) | 1 | - Reducing cost and increasing access to essential medicine | - National Essential Medicines System (NEMS), a policy which aimed to eliminate the profit link between physicians and the pharmaceutical industry, comprising: - *Establishing the National Essential Drugs/Medicines List* - *Sustaining quality standards* - *Running the zero markup policy on essential medicines sales and mandating prescription from the list* - *Improving rational drug use* - *Improving public procurement* | China | Provided an empirical analysis of the demand- and supply-side effects of Shandong’s implementation of the Essential Medications List. | OOPE, Impoverishment |
| (Chen et al., 2020) | 1 | Reducing cost and increasing access to essential medicine | - NEMS (zero markup policy on essential medicines sales and mandating prescription from the list) | China | Evaluated the impacts of the drug markup reduction policy (part of NEMS) on hospital expenditure of esophageal cancer surgery inpatients. | OOPE, Service utilisation, Equity, Other |
| (Y. Chen et al., 2023) | 1 | - Demand side financing | - Medical Financial Assistance subsidizes insurance premiums for low-income households and issues cash transfers for a percentage of OOPE for low-income people after medical insurance reimbursement. | China | Explored the impact of Medical Financial Assistance on healthcare expenses and financial burden in rural areas. | CHE |
| (Y.-J. Chen et al., 2023) | 1 | - Provider payment reforms | - Diagnostic Intervention Packet, similar to diagnostic-related groups but excludes demographic factors like age and gender in classifications and determines payments post-treatment in line with regional budgets | China | Evaluated the impact of Diagnostic Intervention Packet payment system on total costs, OOE, length of stay, and quality of care in hospitalized patients of different ages. | OOPE, CHE, Impoverishment |
| (Choudhary et al., 2022) | 1 | - Behaviour change | - Kangaroo mother care (A health care intervention which involved promoting prolonged skin-to-skin contact and exclusive breastfeeding for neonates through nine home visits in the first 28 days, where workers provided guidance, observed practices, and helped families overcome barriers to these practices) | India | Assessed the effect of community-initiated Kangaroo Mother Care on financial risk protection for low birthweight infants. | OOPE, Service utilisation, Equity |
| (Chu et al., 2023) | 1 | - Reducing cost and increasing access to essential medicine | - NEMS (zero markup policy on essential medicines sales and mandating prescription from the list) | China | To assess the effects of the zero markup policy on patient expenditure. | OOPE, Impoverishment, Equity |
| (Cui et al., 2024) | 1 | - Complex | - New Health Care Reform, a comprehensive reform launched in 2009 consisting of health insurance expansion and structural changes on health care providers. | China | Evaluated the impact of the Essential Public Health Service (EPHS) programme among hypertensive patients to provide evidence for programme progress. | OOPE, CHE, Service utilisation |
| (Dai et al., 2016) | 1 | - Expanded benefit package or enhanced compensation | - Social Pooling Account and Household Account within NCMS | China | Explored the impact of additional coverage via NCMS on medical service utilisation and medical expense of inpatients from 2012 to 2014 | OOPE, CHE, Impoverishment, Service utilisation, Equity, Other |
| (Damrongplasit and Atalay, 2021) | 1 | - Provider payment reforms | - Diagnostic-related groups (shift from fee-for-service) | Thailand | Quantified the effects of diagnostic related group-based payment reform on hospital admissions, length of stay, and OOPE. | OOPE, CHE, Equity |
| (Darvishi et al., 2021) | 1 | - Complex | - Health Transformation Plan (also referred to as Health Sector Evolution Plan) | Iran | Systematically investigated the impact of HTP on OOPE and CHE after implementation. | Impoverishment |
| (Diao et al., 2022) | 1 | - Reducing cost and increasing access to essential medicine | - Public health insurance coverage of novel antibreast cancer medicines | China | Mimicked a quasi-experimental design to estimate the impact of the public health insurance coverage policy on all the indicated patients with a before/after comparison of the total breast cancer-associated direct medical expenditures for a standard course of treatment or maintenance treatment and the proportionate patient OOPE based on the real clinical data. | OOPE |
| (Dickerson et al., 2020) | 1 | - User fee reforms | - Removal of user fees for antiretroviral treatment | Malawi | Investigated the impact of treatment access on medical spending, capacity to pay and catastrophic health expenditures. | Impoverishment, Equity |
| (Ding and Wu, 2017) | 1 | - Reducing cost and increasing access to essential medicine | - NEMS | China | Assessed the effects of the National Essential Medicine Policy on outpatient service utilisation and expenditures. | OOPE, CHE, Impoverishment |
| (Dror et al., 2016) | 1 | - VHI | - Implementation of three CBHI schemes introduced by the Micro Insurance Academy in 2010 with local NGOs in Pratapgarh and Kanpur Dehat (Uttar Pradesh) and Vaishali (Bihar), targeting households linked to women’s self-help groups. Coverage varied by district. | India | Analysed the impact of CBHI on self-medication and financial position in rural Uttar Pradesh and Bihar. | CHE, Service utilisation, Equity |
| (van Duinen et al., 2021) | 1 | - User fee reforms | - Free Health Care Initiative (Removal of user fees for pregnant women, lactating mothers and children under five) | Sierra Leone | Investigated CHE and impoverishment related to caesarean sections in Sierra Leone, assessing impact of user fee removal for C-sections. | OOPE, CHE, Impoverishment, Service utilisation, Equity, Other |
| (Edoka et al., 2016) | 1 | - User fee reforms | - Free Health Care Initiative (Removal of user fees for pregnant women, lactating mothers and children under five) | Sierra Leone | Evaluated the impact of the Free Health Care Initiative (FHCI) on maternal and child healthcare utilisation and OOP payments. | OOPE, CHE, Equity |
| (Ensor et al., 2017) | 1 | - Complex | - Multiple concurrent measures: introduction of user fees, introduction of a health equity fund, vouchers (introduced from 2007, cover costs of family planning, antenatal, delivery and postnatal care and reimbursement for transport to reach the facility for these services), CBHI and strengthening of contracting with public and private providers. | Cambodia | Examined the impact of user fee formalisation policies and subsequent reforms on healthcare utilisation and OOPE over time. | OOPE, Service utilisation, Equity |
| (Erus, 2020) | 1 | - Shift in service delivery organisation and funding | - Family Medicine programme in which the government engaged general practitioners as contractual independent family doctors to provide free preventive care and family medicine services, sometimes requiring a co-payment. | Türkiye | Assessed changes in OOPE following co-payment policies and the extension of primary care services. | CHE |
| (Erus and Aktakke, 2012) | 1 | - Provider payment reforms | - Private Hospital Contracts (2004): Social security organisations began contracting with private hospitals, allowing insured members to use private facilities. - Performance-Based Payment Scheme (2004): MoH hospitals adopted a performance-based payment model, similar to fee-for-service, which incentivised providers to increase service volumes, improving hospital productivity. - Hospital Ownership Transfer (2005): management of hospitals was transferred to the MoH, consolidating hospital administration under the Ministry to enhance efficiency and streamline service delivery. | Türkiye | Analysed the impact of several health financing reforms on OOPE for public insurees. | CHE |
| (Esmaeili et al., 2021) | 1 | - Complex | - Health Transformation Plan (also referred to as Health Sector Evolution Plan) | Iran | Evaluated the impact of the Health Transformation Plan on health spending in Iran. | OOPE, Service utilisation, Equity |
| (Fan et al., 2012) | 1 | - PFHI | - Rajiv Aarogyasri Community Health Insurance in Andhra Pradesh State, offered to families living below the poverty line and eligible for the national RSBY scheme | India | Evaluated the impact of the Aarogyasri health insurance programme on the reduction of CHE in households below the poverty line. | Impoverishment, Equity |
| (Fang et al., 2012) | 2 | - SHI - VHI | - Urban Employee Basic Medical Insurance (UEBMI) - URBMI - NCMS started in 2003, thereafter NRCMS, started in 2007 - URRBMI (Urban and Rural Resident Medical Insurance) (integrated URBMI and NRCMS in 2016) - Commercial health insurance: Work-place health insurance which supplements SHI schemes. | China | Described the effect of insurance on the covered population in China, including coverage, gross and out-of-pocket spending, and coping mechanisms. | CHE |
| (Fiestas Navarrete et al., 2019) | 1 | - SHI | - National Health Insurance Scheme which involves voluntary enrolment for persons in the informal sector with an income-dependent premium. | Ghana | Tested the impact of national health insurance on utilisation and financial risk protection compared with non-enrollment. | OOPE |
| (Fink et al., 2013) | 1 | - VHI | - CBHI in which insured members were assigned to one public primary care facility based on geographical location, and were supposed to get free treatment without any co-payment, ceiling or limit on all services included in the scheme’s benefit package. | Burkina Faso | To evaluate the welfare and health impact of the insurance programme. | CHE, Impoverishment, Service utilisation, Equity, Other |
| (Fontanet et al., 2021) | 1 | - Shift in service delivery organisation and funding | - Maternity Waiting Homes (residential facilities located near health care facilities qualified to provide basic emergency obstetric care utilised by expecting mothers within 1-2 weeks of delivery, with a focus on the most remote women) | Zambia | Assessed delivery-related expenditure for women using maternity waiting homes (MWHs) versus rural health facilities. | CHE, Impoverishment |
| (Frimpong et al., 2021) | 1 | - SHI | - National Health Insurance Scheme which involves voluntary enrolment for persons in the informal sector with an income-dependent premium. | Ghana | Estimated the average exit time from CHE and examined the effect of national health insurance on exit time. | OOPE, Equity |
| (Gao et al., 2014) | 1 | - Provider payment reforms | - Insurance fund in URBMI was divided into three parts to reimburse inpatient care: capitation fund, the equalisation fund (meant to compensate the loss of small hospitals), and the preservation fund (created to reimburse providers for performing special outpatient services). | China | Assessed the impact of capitation reform on out-of-pocket costs. | OOPE |
| (Garcia-Diaz et al., 2023) | 1 | - PFHI | - Seguro Popular (voluntary national health insurance scheme to provide health care for the poor) | Mexico | Analysed the long-term effects of the Seguro Popular on OOPE equity in the poor Mexican population. At the time of the study, Seguro Popular had been phased out and replaced by the Health Institute for Welfare (Instituto de Salud para el Bienestar (INSABI)). | OOPE, CHE, Equity |
| (Garg et al., 2019) | 1 | - PFHI | - Broadly examined PFHI in India, which includes RSBY (government health insurance programme launched in 2008 providing hospital-based healthcare services to families living below the poverty line), PMJAY (PFHI launched in 2018), and state-level insurance for poor individuals eligible for the national PFHI scheme | India | Examined the performance of PFHI in providing financial protection for the elderly in India. | OOPE, Service utilisation, Equity, Other |
| (Garg et al., 2020) | 1 | PFHI | - PMJAY (PFHI launched in 2018, replacing RSBY) | India | Examined the effect of enrollment under PMJAY on hospital utilisation and financial protection in Chhattisgarh. | OOPE, Service utilisation, Equity |
| (Garg et al., 2022a) | 1 | - PFHI | - Broadly examined PFHI in India, which includes RSBY (government health insurance programme launched in 2008 providing hospital-based healthcare services to families living below the poverty line), PMJAY (PFHI launched in 2018, replacing RSBY), and state-level insurance for poor individuals eligible for the national PFHI scheme | India | Examined the effects of PFHI on financial protection among the elderly. | CHE, Impoverishment |
| (Garg et al., 2022b) | 1 | - PFHI | - Broadly examined PFHI in India, which includes RSBY (government health insurance programme launched in 2008 providing hospital-based healthcare services to families living below the poverty line), PMJAY (PFHI launched in 2018, replacing RSBY), and state-level insurance for poor individuals eligible for the national PFHI scheme | India | Examined the change in access and financial protection for non-Covid hospitalisations during the Covid-19 pandemic in Chhattisgarh state and examined the performance of PFHI in this context. | OOPE, CHE, Equity |
| (Gopalan and Durairaj, 2012) | 1 | - Demand side financing | - Janani Suraksha Yojana conditional cash transfer to encourage institutional deliveries | India | Explored the impact of a cash transfer programme on financial access to maternal healthcare, OOPS, and provider motivations. | OOPE, Service utilisation, Equity |
| (Gotsadze et al., 2015b) | 1 | - PFHI | - Government-funded medical insurance for the poor | Georgia | Assessed the impact of medical insurance for the poor on service utilisation and financial protection. | OOPE |
| (Gotsadze et al., 2015a) | 1 | - PFHI | - Government-funded medical insurance for the poor | Georgia | Examined whether medical insurance for the poor affected utilisation and costs differently among chronic patients compared to those with acute health needs. | OOPE, Equity, Other |
| (Grogger et al., 2015) | 1 | - PFHI | - Seguro Popular (voluntary national health insurance scheme to provide health care for the poor) | Mexico | Estimated differences in the effect of Seguro Popular within rural areas according to the type of health-care facility to which beneficiaries have access and assessed its effect on CHE in urban areas. | OOPE, Other |
| (Gu et al., 2017) | 1 | - SHI | - NCMS started in 2003, thereafter NRCMS, started in 2007 | China | Measured the incidence of CHE, analysed the income-related inequality in CHE, and explored the role that the NCMS and adjustment of its reimbursement rates play in reducing rural residents' incidence of CHE | OOPE, CHE, Impoverishment, Service utilisation |
| (Guo et al., 2016) | 1 | - SHI | - NCMS started in 2003, thereafter NRCMS, started in 2007 | China | This study aims to examine whether the NCMS and its recent expansion have reached the goal of reducing the risk and inequality of catastrophic health spending for rural residents in China | OOPE, Service utilisation |
| (Hasan et al., 2024) | 1 | - PFHI | - Shasthyo Surokhsha Karmasuchi (government-initiated social health protection scheme for people living below the poverty line) | Bangladesh | Assessed the effect of Shasthyo Surokhsha Karmasuchi (SSK) on OOPE, CHE, and impoverishment for the enrolled population in Tangail District, Bangladesh. | OOPE, Service utilisation, Equity |
| (He and Nolen, 2019) | 1 | - SHI | - UEBMI | China | Examined the effect of UEBMI on insurance coverage, health service use, preventative care, and OOP expenditure. | OOPE, Equity |
| (He et al., 2017) | 1 | - Provider payment reforms | - Shift from fee-for-service to global budget reforms | China | Analysed the effects of global budget reform in areas where it has been implemented in China. | OOPE, Equity |
| (Homaie Rad et al., 2017) | 1 | - Shift in service delivery | - Family Physician plan which involves a general practitioner leading a team that provides PHC, preventive care, and referrals, and introduces a gatekeeping function to referrals. Launched in 2005 for rural population, extended in 2011 for urban population. | Iran | Evaluated the effects of Iran's urban family physician plan during a period of national economic instability, examining the effect of an economic crisis on the healthcare program. | OOPE, Service utilisation, Equity |
| (Honda and Hanson, 2013) | 1 | - Reducing cost and increasing access to essential medicine | - Equity funds‚ implemented at public health centres to subsidize free medicine for the poor | Madagascar | Assessed the equity funds in Madagascar from three perspectives: accuracy of targeting; improvement in health care access for the poor; and reduction in financial burden on the poor. | OOPE, CHE, Equity, Other |
| (Hu et al., 1999) | 1 | - SHI | - Labour Medical Insurance System (implemented in 1951 to cover the employees of state-owned or collectively-owned enterprises as well as their dependents. Was replaced by the UEBMI in 1998.) | China | Analysed the impact of enterprise reform (expansion of Labour Medical Insurance System to include collective-owned enterprises (which are owned by local or district governments), in addition to state-owned enterprises) on workers' health care benefits and their financial burden due to medical expenses. | Other |
| (Huang et al., 2018) | 1 | - Complex | - New Health Care Reform, a comprehensive reform launched in 2009 consisting of health insurance expansion and structural changes on health care providers. | China | To examine the availability, use, and affordability of medicines in urban China following the 2009 Health Care System Reform. | OOPE, Equity |
| (Huang et al., 2023) | 1 | - Complex | - Maternal and Child Health Policies and Programs for Poverty Alleviation (series of policies and programs that expanded services, infrastructure, workforce support, and financial protection for women and children, implemented from 2015 to 2020) | China | Assessed the changes in disparities in maternal and child health service coverage and health outcomes between poor and non-poor areas at township level before and after the Maternal and Child Health Policies and Programs for Poverty Alleviation. | OOPE, Service utilisation, Equity |
| (Huo et al., 2023) | 1 | - Insurance integration/ overarching assessment of multiple schemes | - URRBMI (integrated URBMI and NRCMS in 2016) | China | Examined the effect of urban and rural medical insurance integration on medical impoverishment in China. | OOPE, CHE, Impoverishment, Service utilisation, Equity, Other |
| (J. Jiang et al., 2019) | 1 | - Expanded benefit package or enhanced compensation | - Critical illness Insurance | China | Determined the impact of critical illness insurance on reducing financial burden and improving health equity. | OOPE, Equity, Other |
| (W.-X. Jiang et al., 2019) | 1 | - Provider payment reforms | - Case-based payment reform (from fee-for-service) increasing the reimbursement rate for TB patients | China | Investigated changes in OOPE and financial burden on TB patients following implementation of case-based payment, focusing on differential impacts by income group. | OOPE, Service utilisation, Equity |
| (Jiang et al., 2021) | 1 | - SHI | - URRBMI (integrated URBMI and NRCMS in 2016) | China | To explore the perceived financial risk protection effect of URRBMI and its influencing factors | Service utilisation, Other |
| (Jing et al., 2013) | 1 | - Expanded benefit package or enhanced compensation | - NCMS started in 2003, thereafter NRCMS, started in 2007 enhanced compensation and extended benefit package (focused initially on increasing reimbursement ratio for inpatient services, thereafter started to cover outpatient services and increased reimbursement ratios over time). | China | Evaluated changes in household CHE before and after reimbursement policies for chronic diseases. | CHE, Impoverishment, Equity, Other |
| (Joshani Kheibari et al., 2019) | 1 | - Complex | - Health Transformation Plan (also referred to as Health Sector Evolution Plan) | Iran | Evaluated the 2014-2016 health system reform effects on health payment distribution trends from 2010-2016. | OOPE, CHE, Impoverishment |
| (Jowett et al., 2003) | 1 | - VHI | - Voluntary health insurance in Vietnam covers 11% of the population (as of 2007), financed by individual private premiums based on financial capacity | Vietnam | Compared OOP expenditures between members and eligible non-members of the voluntary health insurance scheme. | CHE, Impoverishment, Equity |
| (Kanmiki et al., 2019) | 1 | - SHI | - National Health Insurance Scheme which involves voluntary enrolment for persons in the informal sector with an income-dependent premium. | Ghana | Assessed the impact of Ghana's national health insurance scheme on OOP payments. | OOPE, CHE, Impoverishment |
| (Karunaratna et al., 2019) | 1 | - SHI | - Agrahara (mandatory SHI for public sector employees) | Sri Lanka | Examined healthcare utilisation patterns among public sector employees and analysed the impact of Agrahara SHI on reducing illness-related financial burden. | OOPE, Service utilisation, Equity |
| (Knaul et al., 2018) | 1 | - PFHI | - Seguro Popular (voluntary national health insurance scheme to provide health care for the poor) | Mexico | To determine the impact of Seguro Popular on CHE, impoverishing household expenditures and on the financial protection of the Mexican health system. | Impoverishment |
| (Koch et al., 2022) | 1 | - SHI | - Compulsory CBHI (Mutuelles) which covers a basic package of services and drugs from authorised providers and for which Ubudehe Category 1 patients do not pay any annual premium or copay | Rwanda | Measured the economic burden of cesarean sections and the financial protection provided by CBHI. | OOPE, Equity |
| (Korachais et al., 2019) | 1 | - User fee reforms | - User fee exemption of health centre outpatient consultations for the poor as part of Health Equity Fund | Cambodia | Evaluated the impact of the extension of the Health Equity Fund to health centre outpatient services on health-seeking behaviour and related health expenditure. | OOPE, CHE, Service utilisation, Equity, Other |
| (Kundu et al., 2018) | 1 | - Expanded benefit package or enhanced compensation | - Inclusion of MDR-TB benefit packages as part of the universal health insurance scheme | India | Investigated the implementation gap between policy and practice of MDR-TB benefit packages in Chhattisgarh, focusing on equity. | OOPE, Impoverishment, Service utilisation, Equity, Other |
| (Lépine et al., 2018) | 1 | - User fee reforms | - Removal of user fees in public primary healthcare facilities (rural areas in 2006, urban areas in 2011) | Zambia | Evaluated the impact of user fee removal on healthcare utilisation and OOP expenditures. | OOPE, CHE |
| (Lambert-Evans et al., 2009) | 1 | - User fee reforms | - Removal of user fees for indigents | Burundi | Evaluated the exemption mechanism providing free healthcare for indigents in Karuzi province, Burundi. | OOPE, Service utilisation |
| (Lei and Lin, 2009) | 1 | - SHI | - NCMS started in 2003, thereafter NRCMS, started in 2007 | China | This study explores the impact of the NCMS, a newly adopted public health insurance programme in rural China | OOPE, Equity, Other |
| (Li et al., 2015) | 1 | - Expanded benefit package or enhanced compensation | - NCMS started in 2003, thereafter NRCMS, started in 2007 enhanced compensation and extended benefit package (focused initially on increasing reimbursement ratio for inpatient services, thereafter started to cover outpatient services and increased reimbursement ratios over time). | China | Evaluated the impact of NRCMS on impoverishment due to major health hazards, financial risks, and rural income equity. | CHE, Impoverishment, Equity |
| (Li et al., 2018) | 1 | - Provider payment reforms | - Implementation of quota payment system reform as part of NRCMS global budget reform | China | Assessed the quota payment system reform for specific diseases under a global budget in rural China, as part of the NRCMS. | OOPE, CHE, Impoverishment, Service utilisation, Equity, Other |
| (J. Li et al., 2019) | 1 | - Expanded benefit package or enhanced compensation | - NCMS started in 2003, thereafter NRCMS, started in 2007 enhanced compensation and extended benefit package (focused initially on increasing reimbursement ratio for inpatient services, thereafter started to cover outpatient services and increased reimbursement ratios over time). | China | Investigated changes in the NCMS (benefit expansion to cover outpatient expenditure) on medical expenditures of elderly rural migrants. | OOPE, Service utilisation, Equity |
| (L. Li et al., 2019) | 1 | - Expanded benefit package or enhanced compensation | - Critical illness Insurance | China | Evaluated the impact of Critical illness insurance on the burden of high-cost groups in central rural China. | Impoverishment, Service utilisation, Equity |
| (A. Li et al., 2019) | 1 | - Expanded benefit package or enhanced compensation | - Critical Illness Insurance | China | Evaluated the extent of CHE in Jiangsu Province and the impact of Critical Illness Insurance on CHE and associated factors. | OOPE, Service utilisation |
| (H. Li et al., 2019) | 1 | - Provider payment reforms | - Implementation of integrated payment system reform as part of NRCMS global budget reform | China | Evaluated the effect of integrated payment system reform, as part of the New Rural Cooperative Medical System (NRCMS), on economic burden and readmission rates for cerebral infarction inpatients. | OOPE, Service utilisation, Equity, Other |
| (Li et al., 2020) | 1 | - Expanded benefit package or enhanced compensation | - Serious Illness Medical Insurance (SIMIS) (affiliated with the NRCMS and URBMI as complementary medical insurance) | China | Analysed the effect of the implementation of serious illness medical insurance on alleviating the economic burden of rural residents in Jinzhai County | OOPE, CHE, Equity |
| (Zhipeng Li et al., 2023) | 1 | - Complex | - Health Poverty Alleviation Programme (HPAP) is a multi-component poverty alleviation programme that strengthened health insurance and medical assistance for poor rural households, improved targeting through dynamic data systems, promoted equitable urban–rural resource distribution, upgraded healthcare infrastructure, expanded telemedicine, and incentivised the deployment of health workers to underserved areas. | China | Examined HPAP’s enduring impact on poverty reduction and potential moral hazard. | OOPE, CHE, Service utilisation, Equity, Other |
| (Zhihui Li et al., 2023) | 1 | - Social protection | - Disability-targeted cash transfer programme for disabled adults in difficulties and for carers of People with Severe Disabilities (CT-PSD) | China | Investigated the association between China’s disability-targeted cash transfer programme and disability status, as well as equitable access to rehabilitation and medical services | Impoverishment, Service utilisation, Equity |
| (Liu et al., 2017) | 2 | - Demand side financing - SHI | - Medical Financial Assistance subsidizes insurance premiums for low-income households and issues cash transfers for a percentage of OOPE for low-income people after medical insurance reimbursement. | China | Investigated associations between Medical Financial Assistance subvention for SHI enrollment, Medical Financial Assistance cash aid and CHE, and SHI enrollment and CHE in low-income households. | OOPE, Equity |
| (Liu et al., 2021) | 1 | - Complex | - New Health Care Reform, a comprehensive reform launched in 2009 consisting of health insurance expansion and structural changes on health care providers. | China | Examined how multiple healthcare reforms affected catastrophic health spending of low- income households in China. | OOPE, CHE, Service utilisation |
| (K. Liu et al., 2023) | 1 | - SHI | - UEBMI | China | Examined the impacts of China's UEBMI on OOPE of individuals. | OOPE, Service utilisation, Equity, Other |
| (Y. Liu et al., 2023) | 1 | - Reducing cost and increasing access to essential medicine | - NEMS (National Essential Drugs/Medicines List) | China | To evaluate the implementation effect of hepatitis C medical insurance reimbursement policy in China from the view of medical institutions | OOPE, Service utilisation, Equity |
| (Lu et al., 2012) | 1 | - SHI | - Compulsory CBHI (Mutuelles) which covers a basic package of services and drugs from authorised providers and for which Ubudehe Category 1 patients do not pay any annual premium or copay | Rwanda | Evaluated the impact of Mutuelles on achieving universal healthcare coverage and financial risk protection in its first eight years. | CHE |
| (Ly et al., 2022) | 2 | - VHI - PFHI | - Voluntary CBHI scheme that includes a general scheme (CBHI-1), which is partially subsidised, and a special scheme for the poor (CBHI-2), which is fully financed by the state (registration and free care). | Senegal | Assessed the impact of subsidized community health insurance in Senegal, particularly on the poor. | OOPE, CHE, Service utilisation, Other |
| (Ma and Xu, 2022) | 1 | - VHI | - Long Term Care Insurance | China | Examined whether Long-Term Care Insurance reduces OOPE and aligns with value-based healthcare. | OOPE |
| (Ma et al., 2016) | 1 | - SHI | - NCMS started in 2003, thereafter NRCMS, started in 2007 | China | Examined the protection effectiveness of NCMS by analysing the health care utilisation and medical expenditures, and investigated the protection fairness by comparing the changes in the prevalence of CHE and concentration indices among different income-level household groups, pre- and post-NCMS reimbursement. |  |
| (Ma et al., 2021) | 1 | - SHI | - NCMS started in 2003, thereafter NRCMS, started in 2007 | China | Estimated the economic burden of cardiovascular disease (CVD) and identified weaknesses in the design of medical insurance. | OOPE, Service utilisation |
| (Malekroudi et al., 2023) | 1 | - Complex | - Health Transformation Plan (also referred to as Health Sector Evolution Plan) | Iran | Researched the Health Transformation Plan’s effects on financial outcomes of physiotherapy services, including OOPE and CHE. | CHE, Equity |
| (Maroof and Sangmi, 2021) | 1 | - PFHI | - RSBY (government health insurance programme providing hospital-based healthcare services to families living below the poverty line) | India | Assessed the financial impact of the health insurance programme for the poor. | OOPE, CHE, Impoverishment, Service utilisation, Equity, Other |
| (Martínez-García et al., 2018) | 1 | - PFHI | - Seguro Popular (voluntary national health insurance scheme to provide health care for the poor) | Mexico | The study aimed to evaluate the impact of the Popular Insurance (PI) programme in Mexico on catastrophic medical expenses associated with ST-segment elevation myocardial infarction (STEMI).  It is desirable to study the effects of PI to improve our understanding of how the reduction of out-of-pocket spending impacts people's lives. Here, we analysed the impact of the PI in three areas: (1) hospital care management, (2) costs associated with treatment, and (3) main outcomes such as hospital readmission, mortality, and therapeutic adherence. This study aims to address the extent and effect of this governmental strategy to cover STEMI catastrophic expenses at the National Institute of Cardiology, Mexico. | OOPE, CHE, Impoverishment, Service utilisation, Equity, Other |
| (Masiye et al., 2016) | 1 | - User fee reforms | - Removal of user fees in public primary healthcare facilities (rural areas in 2006, urban areas in 2011) | Zambia | Examined financial protection from fees following the decision to abolish user fees in public primary healthcare facilities. | OOPE, CHE, Impoverishment, Service utilisation, Equity, Other |
| (Mebratie et al., 2019) | 1 | - VHI | - Voluntary, government-run CBHI scheme with community involvement in scheme design, management and supervision | Ethiopia | Identified the impact of a voluntary CBHI scheme on healthcare utilisation, cost of care and out-of-pocket expenditure. | OOPE, CHE, Impoverishment, Service utilisation, Equity, Other |
| (Meng et al., 2022) | 1 | - Provider payment reforms | - Two-stage funding reform in county hospitals: Diagnostic-related group-based payments for inpatient care and capitation funding for outpatient care | China | Evaluated the associations of the Chinese diagnosis-related group system with inpatient expenditures for older adults with hip fractures. | OOPE, CHE, Impoverishment, Service utilisation, Equity, Other |
| (Meng et al., 2019b) | 1 | - Provider payment reforms | - Episode-based bundled payment for childbirth (shifting from fee-for-service) | China | Investigated the effects of episode-based bundled payment on cesarean delivery rates and childbirth costs in rural China. | OOPE, CHE, Impoverishment, Service utilisation, Equity, Other |
| (Meng et al., 2019a) | 1 | - Provider payment reforms | - Systemic hospital payment reform in Sanming implemented two-invoice drug procurement, prescription monitoring, price adjustments, and mixed fee-for-service and diagnostic-related group payments to curb costs and inefficiency | China | Analysed the systemic reform's impact on the financial burden and length of stay for the rural population in Sanming. | OOPE, CHE, Impoverishment, Service utilisation, Equity, Other |
| (Miao et al., 2018) | 1 | - Expanded benefit package or enhanced compensation | - NCMS started in 2003, thereafter NRCMS, started in 2007 enhanced compensation and extended benefit package (focused initially on increasing reimbursement ratio for inpatient services, thereafter started to cover outpatient services and increased reimbursement ratios over time). | China | Estimated the effects of SHI reforms on medical utilisation, expenditures, reimbursement, and health outcomes. | OOPE, CHE, Impoverishment, Service utilisation, Equity, Other |
| (Mohammadzadeh et al., 2023) | 1 | - Complex | - Health Transformation Plan (also referred to as Health Sector Evolution Plan) - Targeted Subsidy Plan, launched in December 2010 as part of a broader Iranian economic reform replaced energy and food subsidies with targeted social assistance | Iran | Examined the impact of two government programmes (Health Transformation Plan, and the targeted subsidy plan) on equity in health financing. | OOPE, CHE, Impoverishment, Service utilisation, Equity, Other |
| (Serván-Mori et al., 2023) | 1 | - PFHI | - Instituto de Salud para el Bienestar (INSABI) (voluntary national health insurance scheme established in January 2020 to replace Seguro Popular, aiming to improve coverage and quality amid corruption concerns) | Mexico | Examined the impact of health policy changes (dismantling of Seguro Popular and introduction of Instituto de Salud para el Bienestar (INSABI), and the COVID-19 pandemic on financial risk protection in Mexico (2018-2020). | OOPE, CHE, Impoverishment, Service utilisation, Equity, Other |
| (Mori et al., 2024) | 1 | - Social protection | - Cash transfers to girls and their parents | Zambia | Evaluated the impact of cash support programmes on healthcare utilisation and CHE among households participating in a cluster-randomized controlled trial focusing on adolescent childbearing in rural Zambia. | OOPE, CHE, Impoverishment, Service utilisation, Equity, Other |
| (Mukherjee and Singh, 2018) | 1 | - Demand side financing | - Janani Suraksha Yojana conditional cash transfer to encourage institutional deliveries | India | Examined the extent to which a cash transfer programme reduces catastrophic expenditure on maternity care and the adequacy of incentives to cover household costs. | OOPE, CHE, Impoverishment, Service utilisation, Equity, Other |
| (Nannini et al., 2021) | 1 | - Demand side financing | - Community based zero-interest health loans | Uganda | Assessed the impact of a pilot with zero-interest loans on health expenditures in rural Uganda. | OOPE, CHE, Impoverishment, Service utilisation, Equity, Other |
| (Neelsen and O’Donnell, 2017) | 1 | - User fee reforms | - Full state-funded subsidies at public facilities for poor adult population | Peru | Study analyses the impact of granting the poor entitlement to tax-financed basic care without charge by comparing changes in utilisation with those already covered through employment-based insurance. | OOPE, CHE, Impoverishment, Service utilisation, Equity, Other |
| (Nemati et al., 2020) | 1 | - Complex | - Health Transformation Plan (also referred to as Health Sector Evolution Plan) | Iran | Assessed OOP payments and CHE exposure among households in Tabriz after the Health Transformation Plan implementation. | OOPE, CHE, Impoverishment, Service utilisation, Equity, Other |
| (Ng et al., 2024) | 1 | - VHI | - Supplementary/voluntary private health insurance | Malaysia | Investigated the effect of supplementary private health insurance on OOPE for inpatient care in Malaysia. | OOPE, CHE, Impoverishment, Service utilisation, Equity, Other |
| (Nguyen, 2016) | 2 | - VHI - PFHI | - Voluntary Student Health Insurance (for school children) - Health Care Fund for the Poor (HCFP), offering free health insurance for poor children, and children in target groups | Vietnam | Examined the impact of student health insurance and free health insurance on the health care utilisation and health expenditures of children from 6 to 14 years old in Vietnam. | OOPE, CHE, Impoverishment, Service utilisation, Equity, Other |
| (Nguyen, 2020) | 1 | - SHI | - Vietnam Social Security, which integrated all health insurance pools and expanded publicly funded health insurance | Vietnam | Evaluated the effects of public health insurance expansion on healthcare utilisation, OOP expenditures, and health outcomes in nonpoor young children in Vietnam. | OOPE, CHE, Impoverishment, Service utilisation, Equity, Other |
| (Nguyen and Lo Sasso, 2019) | 1 | SHI | Community Health Insurance covers inpatient treatment at state hospitals and outpatient services but only includes inpatient drugs on a Ministry of Health list, typically excluding outpatient medications. It  comprises three programs covering 41% of the population as of 2007:   - Social Health Insurance: Covers 9% of the formally employed, retirees, and dependents of military and police officers, funded by a payroll tax of up to 3%. - Health Care Fund for the Poor: Covers 18% of the population and receives three-quarters of its funding from the central government. - Free Health Care for Children Under 6: Covers 11% of children, funded entirely by the central government. | Vietnam | Investigated the effects of the 2005 universal health insurance programme for children under age 6 on healthcare utilisation, OOP spending, and health outcomes. | OOPE, CHE, Impoverishment, Service utilisation, Equity, Other |
| (Nguyen and Wang, 2013) | 1 | SHI | Community Health Insurance covers inpatient treatment at state hospitals and outpatient services but only includes inpatient drugs on a Ministry of Health list, typically excluding outpatient medications. It  comprises three programs covering 41% of the population as of 2007:   - Social Health Insurance: Covers 9% of the formally employed, retirees, and dependents of military and police officers, funded by a payroll tax of up to 3%. - Health Care Fund for the Poor: Covers 18% of the population and receives three-quarters of its funding from the central government. - Free Health Care for Children Under 6: Covers 11% of children, funded entirely by the central government. | Vietnam | Evaluated the effects of the Free Care for Children Under Six policy on healthcare utilisation, OOP expenditures, and health outcomes. | OOPE, CHE, Impoverishment, Service utilisation, Equity, Other |
| (Nguyen et al., 2011) | 1 | - SHI | - National Health Insurance Scheme which involves voluntary enrolment for persons in the informal sector with an income-dependent premium. | Ghana | Evaluated the impact of the national health insurance on household OOP and CHE. | OOPE, CHE, Impoverishment, Service utilisation, Equity, Other |
| (K. T. Nguyen et al., 2012) | 3 | - SHI - VHI - PFHI | Community Health Insurance covers inpatient treatment at state hospitals and outpatient services but only includes inpatient drugs on a Ministry of Health list, typically excluding outpatient medications. It  comprises three programs covering 41% of the population as of 2007:   - Social Health Insurance: Covers 9% of the formally employed, retirees, and dependents of military and police officers, funded by a payroll tax of up to 3%. - Health Care Fund for the Poor: Covers 18% of the population and receives three-quarters of its funding from the central government. - Free Health Care for Children Under 6: Covers 11% of children, funded entirely by the central government. | Vietnam | Assessed the effects of several reformed health insurance schemes on healthcare treatment and costs for households. | OOPE, CHE, Impoverishment, Service utilisation, Equity, Other |
| (H. T. H. Nguyen et al., 2012) | 1 | - Demand side financing | - Voucher programme involving cash incentives and free access to antenatal, delivery, and postnatal care, as well as cash incentives for providers to offer these services. | Bangladesh | Evaluated the effects of a pilot voucher programme on utilisation of maternal health services and associated OOP expenditures in Bangladesh. | OOPE, CHE, Impoverishment, Service utilisation, Equity, Other |
| (Nguyen et al., 2023) | 1 | - SHI | - Vietnam Social Security, which integrated all health insurance pools and expanded publicly funded health insurance | Vietnam | Examined mechanisms under which an amendment expanding private health insurance eligibility affected enrollment, healthcare utilisation, and OOPE by middle- and low-income households in this transitioning process. | OOPE, CHE, Impoverishment, Service utilisation, Equity, Other |
| (Nikoloski and Mossialos, 2018) | 1 | - PFHI | - Seguro Popular (voluntary national health insurance scheme to provide health care for the poor) | Mexico | Analyzed the medium and longer-range effects of Seguro Popular on out-of-pocket healthcare spending, catastrophic spending, and impoverishment, with the aim of understanding the effect of social security programmes on these factors. | OOPE, CHE, Impoverishment, Service utilisation, Equity, Other |
| (Obare et al., 2015) | 1 | - Demand side financing | - Voucher Programme for subsidised reproductive health services | Kenya | Examined changes in the likelihood of paying OOP and amounts paid for family planning, antenatal care, delivery, and postnatal care services at health facilities over time among women in voucher and non-voucher sites. | OOPE, CHE, Impoverishment, Service utilisation, Equity, Other |
| (Okoroh et al., 2020) | 1 | - SHI | - National Health Insurance Scheme which involves voluntary enrolment for persons in the informal sector with an income-dependent premium. | Ghana | Tested the hypothesis that insurance protects surgical patients from financial catastrophe. | OOPE, CHE, Impoverishment, Service utilisation, Equity, Other |
| (Okunogbe et al., 2022) | 1 | - VHI | - Voluntary CBHI programme that subsidised the cost of annual premiums for enrollees and upgraded selected health facilities in the intervention areas. Enrolment in the insurance programme was at the individual level and could occur on a monthly basis. Coverage lasted for a full year, after which it needed to be renewed. Enrolment and renewal were voluntary. | Nigeria | Examined short- and long-term effects of a health insurance programme in Kwara State on CHE. | OOPE, CHE, Impoverishment, Service utilisation, Equity, Other |
| (Oyando et al., 2023) | 1 | - Expanded benefit package or enhanced compensation | - Inclusion of non-communicable disease services in the National Health Insurance Fund for households with hypertension and/or diabetes for 12 month | Kenya | Measured the share of household expenditure on healthcare, incidence of CHE, and effectiveness of the National Health Insurance Fund in protecting households with hypertension/diabetes in Kenya. | OOPE, CHE, Impoverishment, Service utilisation, Equity, Other |
| (Pan et al., 2022) | 1 | - Demand side financing | - Medical fee direct reimbursement | China | Evaluated the impact of China’s policy on direct settlement of intra-provincial medical reimbursements on financial protection for people aged 45+. | OOPE, CHE, Impoverishment, Service utilisation, Equity, Other |
| (Parmar et al., 2023) | 1 | PFHI | - PMJAY (PFHI launched in 2018, replacing RSBY) | India | Examined how PM-JAY has affected hospitalisations and OOP across public and private facilities. | OOPE, CHE, Impoverishment, Service utilisation, Equity, Other |
| (Peng and Zhu, 2021) | 2 | - SHI - VHI | - UEBMI - URBMI - NCMS started in 2003, thereafter NRCMS, started in 2007 - Private health insurance | China | Compared the impacts of public and private health insurance on financial barriers for people with chronic diseases. | OOPE, CHE, Impoverishment, Service utilisation, Equity, Other |
| (Peresu et al., 2024) | 1 | - Shift in service delivery organisation and funding | - Medicins Sans Frontieres -supported home based MDR-TB project (shift from government led clinic base care) | Eswatini | Assessed costs and treatment success of clinic-based versus home-based MDR-TB care strategies. | OOPE, CHE, Impoverishment, Service utilisation, Equity, Other |
| (Qiu et al., 2014) | 1 | - Reducing cost and increasing access to essential medicine | - NEMS (National Essential Drugs/Medicines List) | China | Described medical costs and utilisation rates of antiviral therapy for chronic hepatitis B (CHB) patients and explored the impact of a partial reimbursement policy. | OOPE, CHE, Impoverishment, Service utilisation, Equity, Other |
| (Rabbani et al., 2022) | 1 | - SHI | - Health Security Scheme (Mandatory employer sponsored insurance for artisans) | Bangladesh | Evaluated the impact of health insurance pilots (Health Security Scheme) on utilisation, OOP expenditures, and well-being among rural women artisans in Bangladesh. | OOPE, CHE, Impoverishment, Service utilisation, Equity, Other |
| (Ranjan et al., 2018) | 1 | - PFHI | - Broadly examined PFHI in India, which includes RSBY (government health insurance programme providing hospital-based healthcare services to families living below the poverty line) and state-level insurance for poor individuals eligible for the national PFHI scheme | India | Assessed the coverage and effectiveness of government purchasing through insurance and tax-funded care for financial protection, PFHI's role in reducing CHE, and the equity dimensions of these strategies. | OOPE, CHE, Impoverishment, Service utilisation, Equity, Other |
| (Raza et al., 2016) | 1 | - VHI | - Implementation of three CBHI schemes introduced by the Micro Insurance Academy in 2010 with local NGOs in Pratapgarh and Kanpur Dehat (Uttar Pradesh) and Vaishali (Bihar), targeting households linked to women’s self-help groups. Coverage varied by district. | India | Evaluated the effects of three CBHI schemes on healthcare utilisation and expenditure. | OOPE, CHE, Impoverishment, Service utilisation, Equity, Other |
| (Ridde et al., 2015) | 1 | - User fee reforms | - Removal of user fees for delivery and children’s healthcare | Burkina Faso | Evaluated the equity impact of the reduction and removal of user fees for the poorest on household OOP expenditures in Burkina Faso. | OOPE, CHE, Impoverishment, Service utilisation, Equity, Other |
| (Riumallo-Herl and Aguila, 2019) | 1 | - Social protection | - Reconocer pension programme, which provided monthly food basket and cash transfers (non-healthcare specific) to adults aged 70 and older | Mexico | To examine the effects of supplemental income to older adults on healthcare use patterns, expenditures and insurance uptake in Yucatan, Mexico | OOPE, CHE, Impoverishment, Service utilisation, Equity, Other |
| (Ruiz et al., 2007) | 1 | - Insurance integration/ overarching assessment of multiple schemes | - Social health insurance including payroll linked insurance, and implementing a subsidised insurance programme for the poorest populations | Colombia | This study was designed to collect and discuss empirical evidence of the effects of the social insurance policy adopted in the Colombian health reform. | OOPE, CHE, Impoverishment, Service utilisation, Equity, Other |
| (Sarkodie, 2021) | 1 | - SHI | - National Health Insurance Scheme which involves voluntary enrolment for persons in the informal sector with an income-dependent premium. | Ghana | Investigated the effect of national health insurance on healthcare utilisation and OOPE from providers' perspectives. | OOPE, CHE, Impoverishment, Service utilisation, Equity, Other |
| (Savitha and K B, 2015) | 1 | - VHI | - Sampoorna Suraksha Programme CBHI | India | Evaluated the impact of Sampoorna Suraksha Program, a nongovernmental organization-initiated scheme, and the risk coping strategies of households faced with medical illness in Karnataka state, India. | OOPE, CHE, Impoverishment, Service utilisation, Equity, Other |
| (Savitha and Kiran, 2015) | 1 | - VHI | - Sampoorna Suraksha Programme CBHI | India | Assessed the effect of the Sampoorna Suraksha Programme on financial protection, including OOPE and CHE. | OOPE, CHE, Impoverishment, Service utilisation, Equity, Other |
| (Shi et al., 2010) | 2 | - Demand side financing - SHI | - Medical Financial Assistance subsidizes insurance premiums for low-income households and issues cash transfers for a percentage of OOPE for low-income people after medical insurance reimbursement. - NCMS started in 2003, thereafter NRCMS, started in 2007 | China | Documented NCMS and Medical Financial Assistance coverage and assessed their effects on access to care and financial protection. | OOPE, CHE, Impoverishment, Service utilisation, Equity, Other |
| (Somkotra and Lagrada, 2008) | 1 | - SHI | - Universal coverage scheme which provides comprehensive outpatient and inpatient care at registered primary care units. The scheme also employed capitation for outpatient care and a global budget with a diagnostic-related group for inpatient care. Some beneficiaries are exempted from the co-payment. | Thailand | Measured household OOP payments for healthcare and examined financial catastrophe and impoverishment during the transition to Universal Coverage (UC). | OOPE, CHE, Impoverishment, Service utilisation, Equity, Other |
| (Sood et al., 2014) | 1 | - PFHI | - Vajpayee Arogyashree scheme in Karnataka State, offered to families living below the poverty line and eligible for the national RSBY scheme | India | Evaluated the effects of Karnataka’s Vajpayee Arogyashree tertiary care insurance programme on OOPE, hospital use, and mortality. | OOPE, CHE, Impoverishment, Service utilisation, Equity, Other |
| (Sosa-Rubí et al., 2011) | 1 | - PFHI | - Seguro Popular (voluntary national health insurance scheme to provide health care for the poor) | Mexico | Estimated the effect of Seguro Popular on the incidence of CHE and OOPE health expenditure in the medium term (6 years after implementation). | OOPE, CHE, Impoverishment, Service utilisation, Equity, Other |
| (Sparrow et al., 2013) | 1 | - SHI | - Health insurance for poor people (Askeskin) | Indonesia | Analysed the impact and targeting effectiveness of the Askeskin subsidized SHI programme. | OOPE, CHE, Impoverishment, Service utilisation, Equity, Other |
| (Sriram and Khan, 2020) | 1 | - PFHI | - RSBY (government health insurance programme providing hospital-based healthcare services to families living below the poverty line) - State-run public health insurance programs for the poor (Chief Minister’s Comprehensive Health Insurance Scheme in Tamil Nadu State, Rajiv Aarogyasri Community Health Insurance in Andhra Pradesh State, and Vajpayee Aarogyasri Scheme in Karnataka State) | India | Examined the effect of insurance programmes for the poor on hospitalisations and inpatient OOPE by comparing those enrolled against a control group of those not enrolled in such programmes. | OOPE, CHE, Impoverishment, Service utilisation, Equity, Other |
| (Sun et al., 2009) | 1 | - SHI | - NCMS started in 2003, thereafter NRCMS, started in 2007 | China | Measured the impact of NCMS on catastrophic payments among rural households in Shandong Province. | OOPE, CHE, Impoverishment, Service utilisation, Equity, Other |
| (Sun et al., 2015) | 1 | - Reducing cost and increasing access to essential medicine | - NEMS | China | Compared the added risk protection offered by NEMS (in addition to the existing NCMS programme) for patients with cardiovascular diseases in rural China. | OOPE, CHE, Impoverishment, Service utilisation, Equity, Other |
| (Sun et al., 2016) | 1 | - SHI | - NCMS started in 2003, thereafter NRCMS, started in 2007 | China | Measured poverty headcount ratio and poverty gap to examine whether NCMS alleviated medical impoverishment in rural Yanbian. | OOPE, CHE, Impoverishment, Service utilisation, Equity, Other |
| (Sun et al., 2021) | 1 | - SHI | - NCMS started in 2003, thereafter NRCMS, started in 2007 - URRBMI (integrated URBMI and NRCMS in 2016) | China | Assessed medical expenditure and CHE among households with lung cancer patients in China following implementation of URBMI and NCMS. | OOPE, CHE, Impoverishment, Service utilisation, Equity, Other |
| (Sunny et al., 2021) | 1 | - User fee reforms | - Free Newborn Care programme | Nepal | To evaluate the change in the OOPE for treatment of sick newborns at hospitals before and after implementation of the Free Newborn Care programme. | OOPE, CHE, Impoverishment, Service utilisation, Equity, Other |
| (Tan et al., 2019) | 1 | - SHI | - UEBMI | China | Examined the impact of SHI generosity on healthcare utilisation and costs. | OOPE, CHE, Impoverishment, Service utilisation, Equity, Other |
| (Tang et al., 2023) | 1 | - Complex | - New Health Care Reform, a comprehensive reform launched in 2009 consisting of health insurance expansion and structural changes on health care providers. | China | Evaluated whether the health system reform improved equity in utilisation and financial burden of health services among patients with hypertension in China | OOPE, CHE, Impoverishment, Service utilisation, Equity, Other |
| (Taverne et al., 2021) | 1 | - VHI | - Voluntary CBHI scheme that includes a general scheme (CBHI-1), which is partially subsidised, and a special scheme for the poor (CBHI-2), which is fully financed by the state (registration and free care) for target groups, including PLHIV. | Senegal | Assessed the capacity of Senegal's health coverage system to reduce health expenditures for PLHIV. | OOPE, CHE, Impoverishment, Service utilisation, Equity, Other |
| (Thein et al., 2021) | 1 | - Provider payment reforms | - Strategic purchasing scheme (Capitation-based health financing) | Myanmar | Assessed the effect of a new capitation-based health financing scheme (Strategic Purchasing clinics) on health service expenditure and financial burden of beneficiary households in Myanmar. | OOPE, CHE, Impoverishment, Service utilisation, Equity, Other |
| (Thornton et al., 2010) | 1 | - VHI | - Seguro Facultativo de Salud (Voluntary health insurance programme for informal sector workers) | Nicaragua | To evaluate determinants of enrolment in a voluntary health insurance programme for informal sector workers and causal effects on expenditures and utilisation. | OOPE, CHE, Impoverishment, Service utilisation, Equity, Other |
| (Thuong et al., 2020) | 2 | - SHI - VHI | - Vietnam Social Security, which integrated all health insurance pools and expanded publicly funded health insurance - Voluntary health insurance | Vietnam | Examined the impact of VHI and heavily subsidized health insurance on healthcare utilisation and OOP expenditures in Vietnam. | OOPE, CHE, Impoverishment, Service utilisation, Equity, Other |
| (Tirgil et al., 2019) | 1 | - PFHI | - Green Card Scheme, giving poor households access to outpatient care, pharmaceuticals and benefits enjoyed by other insured groups (i.e. benefit package expansion/harmonization) | Türkiye | Studied the effects of Green Card scheme expansion on OOP healthcare expenditures among low-income households. | OOPE, CHE, Impoverishment, Service utilisation, Equity, Other |
| (Tirgil et al., 2023) | 1 | - Shift in service delivery organisation and funding | - Family Medicine programme in which the government engaged general practitioners as contractual independent family doctors to provide free preventive care and family medicine services, sometimes requiring a co-payment. | Türkiye | Assessed the impact of the family medicine programme on healthcare expenditures, doctor visits, and hospitalisation costs. | OOPE, CHE, Impoverishment, Service utilisation, Equity, Other |
| (Tripathi et al., 2014) | 1 | - User fee reforms | - Janani Shishu Suraksha Karyakram (user free removal for Delivery, caesarean section, drugs and consumables, diagnostics, diet during stay, provision of blood, and transport between home to health institution during delivery as well as newborn care up to 30 days after birth, offered as a complement to Janani Suraksha Yojana free delivery care in public - health facilities) | India | Assessed the impact of Janani Shishu Suraksha Karyakram on OOPE for childbirth among urban slum dwellers. | OOPE, CHE, Impoverishment, Service utilisation, Equity, Other |
| (Tsuei and Yip, 2024) | 1 | - Provider payment reforms | - Shift from fee-for-service to global budget reforms | - China | Assessed how automony through changed payment system moderated the intervention's effect. | OOPE, CHE, Impoverishment, Service utilisation, Equity, Other |
| (Wagstaff et al., 2009) | 1 | - SHI | - UEBMI - URBMI - NCMS started in 2003, thereafter NRCMS, started in 2007 | China | Estimated the impacts of a health reform project combining supply-side interventions with demand-side measures like Medical Financial Assistance for the poorest. | OOPE, CHE, Impoverishment, Service utilisation, Equity, Other |
| (Wang et al., 2014) | 1 | - SHI | - NCMS started in 2003, thereafter NRCMS, started in 2007 | China | Evaluated the impact of SHI on healthcare utilisation and costs among middle-aged and elderly adults in China. | OOPE, CHE, Impoverishment, Service utilisation, Equity, Other |
| (Wang et al., 2018) | 1 | - SHI | - UEBMI - URBMI - NCMS started in 2003, thereafter NRCMS, started in 2007 | China | Investigated whether a disability-targeted cash transfer programme is associated with improved access to healthcare and education for children and adolescents with disabilities | OOPE, CHE, Impoverishment, Service utilisation, Equity, Other |
| (Wang et al., 2019) | 1 | - Reducing cost and increasing access to essential medicine | - NEMS | China | Examined the long-term effects of the introduction of the Full Coverage of Essential Medicines Policy on the utilisation and accessibility of primary healthcare service for elderly beneficiaries | OOPE, CHE, Impoverishment, Service utilisation, Equity, Other |
| (Wang et al., 2020) | 1 | - Insurance integration/ overarching assessment of multiple schemes | - URRBMI (Urban and Rural Resident Medical Insurance) (integrating URBMI and NCMS in 2016) | China | Assessed whether the URRBMI policy intervention reduces inequity in CHE and identify key determinants of CHE inequity in China | OOPE, CHE, Impoverishment, Service utilisation, Equity, Other |
| (Wang et al., 2023) | 1 | - Social protection | - Disability-targeted cash transfer programme for disabled adults in difficulties and for carers of People with Severe Disabilities (CT-PSD) | China | Investigated whether a disability-targeted cash transfer program was associated with improved access to healthcare and education for children and adolescents with disabilities. | OOPE, CHE, Impoverishment, Service utilisation, Equity, Other |
| (Witter et al., 2016) | 1 | - User fee reforms | - Free C-Section but no other cost (such as complication) covered in Benin - 80% reduction of all fees for deliveries and complications with additional support for transport in Burkina Faso - Free C-Section in Mali - Free delivery and C-Section in Morocco | Benin, Burkina Faso, Mali, Morocco | Analysed the cost and effects of national policies aimed at improving financial access to caesarean and facility deliveries. | OOPE, CHE, Impoverishment, Service utilisation, Equity, Other |
| (Wu and Ercia, 2021) | 2 | - SHI - VHI | - Private health insurance, as complementary to SHI - Urban and rural basic medical insurance schemes in China (URBMI, NRCMS, etc). | China | Examined the expansion of private health insurance within China’s SHI system and its impact on inequities in healthcare utilisation. | OOPE, Service utilisation, Equity |
| (Wu et al., 2022) | 1 | - Provider payment reforms | - Implementation of case-based payment | China | Evaluated the impact of a case-based payment pilot on hospital admissions, treatment patterns, and costs for coronary heart diseases. | OOPE, Other |
| (Xiang et al., 2016) | 1 | - Expanded benefit package or enhanced compensation | - NCMS started in 2003, thereafter NRCMS, started in 2007 enhanced compensation and extended benefit package (focused initially on increasing reimbursement ratio for inpatient services, thereafter started to cover outpatient services and increased reimbursement ratios over time). | China | Measured reimbursement for TB services under the NCMS in rural China and to evaluate changes in CHE caused by the reimbursement policies | OOPE, CHE, Impoverishment, Service utilisation, Equity, Other |
| (Xiang et al., 2024) | 1 | - Provider payment reforms | - Implementation of diagnosis-related group system (from fee-for-service) | China | Examined the impact of diagnostic related group payments on healthcare received by rural residents and informed policymakers on implementation strategies in LMICs. | OOPE, CHE, Impoverishment, Service utilisation, Equity, Other |
| (Xin et al., 2019) | 1 | - Provider payment reforms | - Case-based payment reform (from fee-for-service) increasing the reimbursement rate for TB patients | China | Explored how financial reform affects the financial burden on TB patients through comparison with baseline data. | OOPE, CHE, Impoverishment, Service utilisation, Equity, Other |
| (Xiong et al., 2018) | 1 | - SHI | - UEBMI - URBMI - NCMS started in 2003, thereafter NRCMS, started in 2007 | China | Analysed the impact of Universal Medical Insurance System on medical supply, economic burden, and patient care-seeking behaviour. | OOPE, CHE, Impoverishment, Service utilisation, Equity, Other |
| (Xu et al., 2018) | 1 | - Complex | - New Health Care Reform, a comprehensive reform launched in 2009 consisting of health insurance expansion and structural changes on health care providers. | China | Examined the effect of the New Health Care Reform reform on CHE by comparing the occurrence and inequality of CHE among households with chronic diseases patients before and after the reform | OOPE, CHE, Impoverishment, Service utilisation, Equity, Other |
| (Xu et al., 2019) | 1 | - Complex | - New Health Care Reform, a comprehensive reform launched in 2009 consisting of health insurance expansion and structural changes on health care providers. | China | Investigated the occurrence of impoverishment in urban and rural areas before and after New Health Care Reform (in which PHCs were directed to prescribe drugs only from the National Essential Medicines List and the supplementary provincial essential medicines list), and its determinants. | OOPE, CHE, Impoverishment, Service utilisation, Equity, Other |
| (Xue and Witvorapong, 2023) | 1 | - SHI | - URBMI - UEBMI - NCMS started in 2003, thereafter NRCMS, started in 2007 | China | Investigated the effect of health insurance on healthcare utilisation and health behaviours of older people in China. | OOPE, CHE, Impoverishment, Service utilisation, Equity, Other |
| (Yang and Wu, 2015) | 1 | - Expanded benefit package or enhanced compensation | - NCMS started in 2003, thereafter NRCMS, started in 2007 enhanced compensation and extended benefit package (focused initially on increasing reimbursement ratio for inpatient services, thereafter started to cover outpatient services and increased reimbursement ratios over time). | China | Investigated the impact of NCMS outpatient service coverage expansion on outpatient costs. | OOPE, CHE, Impoverishment, Service utilisation, Equity, Other |
| (Yang et al., 2022) | 1 | - SHI | - UEBMI | China | Examined the horizontal equity of healthcare finance in 2002 and 2007 in a Chinese province. | OOPE, CHE, Impoverishment, Service utilisation, Equity, Other |
| (Yang et al., 2023) | 1 | - Reducing cost and increasing access to essential medicine | - National Drug Price Negotiation policy | China | Examined how national price negotiation and reimbursement policy affect the accessibility of targeted anticancer medicines (TAMs) for cancer patients | OOPE, CHE, Impoverishment, Service utilisation, Equity, Other |
| (Yardim et al., 2014) | 1 | - PFHI | - Green Card Scheme, giving poor households access to outpatient care, pharmaceuticals and benefits enjoyed by other insured groups (i.e. benefit package expansion/harmonization) | Türkiye | Evaluated financial protection and the effects of health reforms on OOP payments during 2003–2009. | OOPE, CHE, Impoverishment, Service utilisation, Equity, Other |
| (You et al., 2016) | 1 | - Expanded benefit package or enhanced compensation | - NCMS started in 2003, thereafter NRCMS, started in 2007 enhanced compensation and extended benefit package (focused initially on increasing reimbursement ratio for inpatient services, thereafter started to cover outpatient services and increased reimbursement ratios over time). | China | Examined the association between the NCMS maternal-services policy adjustment and expense reimbursement in Yuyao, China. | OOPE, CHE, Impoverishment, Service utilisation, Equity, Other |
| (Yu et al., 2013) | 1 | - Reducing cost and increasing access to essential medicine | - NEMS (zero markup policy on essential medicines sales and mandating prescription from the list) | China | Evaluated the impact of full coverage for hypertension drugs (as part of the zero markup policy) on adherence to medication, medical costs, and hypertension control in Shandong Province, China | OOPE, CHE, Impoverishment, Service utilisation, Equity, Other |
| (Yu et al., 2021) | 1 | - Expanded benefit package or enhanced compensation | - Catastrophic Medical Insurance | China | Studied the impact of Catastrophic Medical Insurance on patients' OOPE and health expenditure. | OOPE, CHE, Impoverishment, Service utilisation, Equity, Other |
| (Yuan et al., 2014) | 1 | - Expanded benefit package or enhanced compensation | - NCMS started in 2003, thereafter NRCMS, started in 2007 enhanced compensation and extended benefit package (focused initially on increasing reimbursement ratio for inpatient services, thereafter started to cover outpatient services and increased reimbursement ratios over time). | China | Examined health insurance benefit design impacts on PCI, stent use, inpatient costs, and OOPE for AMI patients. | OOPE, CHE, Impoverishment, Service utilisation, Equity, Other |
| (Zhai et al., 2021) | 1 | - SHI | - NCMS started in 2003, thereafter NRCMS, started in 2007 | China | Assessed the impact of NRCMS on alleviating poverty among rural older adults based on a survey in nine representative counties in western China | OOPE, CHE, Impoverishment, Service utilisation, Equity, Other |
| (Zhang et al., 2016) | 1 | - SHI | - NCMS started in 2003, thereafter NRCMS, started in 2007 | China | Investigated the association between NCMS policy design and its effectiveness in providing financial protection to rural residents. | OOPE, CHE, Impoverishment, Service utilisation, Equity, Other |
| (Zhang et al., 2017) | 1 | - Shift in service delivery organisation and funding | - Public hospital reform involving removed medication mark-up, raised charges for medical services that require professional skills (physician services and surgical fees), and lowered charges for advanced medical diagnostic devices | China | Evaluated the effects of a public hospital pilot project in Hubei province on inpatient spending. | OOPE, CHE, Impoverishment, Service utilisation, Equity, Other |
| (Zhang et al., 2021) | 1 | - Expanded benefit package or enhanced compensation | - NCMS started in 2003, thereafter NRCMS, started in 2007 enhanced compensation and extended benefit package (increased reimbursement ratios and coverage of outpatient services). | China | Assessed the impact of changing NCMS policies on enrollees' service utilisation, medical financial burden and equity between 2003 and 2013. | OOPE, CHE, Impoverishment, Service utilisation, Equity, Other |
| (Zhang et al., 2022) | 1 | - Provider payment reforms | - Implementation of diagnostic-related groups | China | Investigated the impacts of a new payment method in Jinhua on volume, expenditures, and efficiency in Chinese public hospitals. | OOPE, CHE, Impoverishment, Service utilisation, Equity, Other |
| (H. Zhang et al., 2023) | 1 | - Expanded benefit package or enhanced compensation | - Increase of the reimbursement rate and of the cap line and decrease in the deductible line for beneficiaries of the UEBMI | China | Assessed the impact of changes in patient cost-sharing on medical expenses and health outcomes of heart failure patients in Zhejiang province. | OOPE, CHE, Impoverishment, Service utilisation, Equity, Other |
| (T. Zhang et al., 2023) | 1 | - Provider payment reforms | - Two-stage funding reform in county hospitals: Diagnostic-related group-based payments for inpatient care and capitation funding for outpatient care | China | Assessed the effects of a two-stage funding reform on service volume and expenditure at county hospitals in Zhejiang province. | OOPE, CHE, Impoverishment, Service utilisation, Equity, Other |
| (Zhao et al., 2020) | 1 | - Expanded benefit package or enhanced compensation | - Catastrophic Medical Insurance | China | Assessed the effect of catastrophic medical insurance on relieving CHE and impoverishment from catastrophic illnesses in urban and rural China. | OOPE, CHE, Impoverishment, Service utilisation, Equity, Other |
| (Zhong et al., 2021) | 1 | - Expanded benefit package or enhanced compensation | - Critical illness Insurance | China | Examined whether critical illness insurance reduces OOP medical expenditures and improves the effective reimbursement rate in rural China. | OOPE, CHE, Impoverishment, Service utilisation, Equity, Other |
| (Zhou et al., 2017) | 1 | - SHI | - UEBMI - URBMI - NCMS started in 2003, thereafter NRCMS, started in 2007 | China | Assessed the effect of national health insurance on service use and economic burden in urban areas. | OOPE, CHE, Impoverishment, Service utilisation, Equity, Other |
| (Zhou et al., 2022) | 1 | - Insurance integration/ overarching assessment of multiple schemes | - Critical illness insurance - Medical financial assistance - Basic medical insurance in China (NCMS, URBMI, etc) | China | Examined the effect of Triple Medical Security (TMS) (which combines basic medical insurance, critical illness insurance, and medical assistance, targeting the poor with enhanced reimbursement rates and financial support) on poverty in households. | OOPE, CHE, Impoverishment, Service utilisation, Equity, Other |
| (Zhu et al., 2022) | 1 | - Reducing cost and increasing access to essential medicine | - NEMS (zero markup policy on essential medicines sales and mandating prescription from the list) | China | Evaluated the impact of the zero markup policy on healthcare expenditures and utilisation for inpatients. | OOPE, CHE, Impoverishment, Service utilisation, Equity, Other |
| (Zhu et al., 2024) | 1 | - Shift in service delivery organisation and funding | - Investment in PHC via subsidies for public PHC facilities, establishment of a general practitioner led PHC workforce,  programs for postgraduate training and professional development, and improved remuneration and working conditions. | China | Investigated whether strengthening PHC improved health outcomes, financial risk protection, and benefited impoverished groups. | OOPE, CHE, Impoverishment, Service utilisation, Equity, Other |
| (Zoidze et al., 2013) | 1 | - PFHI | - Government-funded medical insurance for the poor | Georgia | Assessed the impact of medical insurance for the poor on equity of access to essential health services and financial protection for targeted and general populations. | OOPE, CHE, Impoverishment, Service utilisation, Equity, Other |
